# Integrated metabolomics and transcriptomics analysis of roots of *Bupleurum chinense* and *B. scorzonerifolium*, two sources of medicinal Chaihu

**DOI:** 10.1038/s41598-022-27019-8

**Published:** 2022-12-26

**Authors:** Hefang Wan, Lei Zhou, Bin Wu, Wenjing Han, Chun Sui, Jianhe Wei

**Affiliations:** Institute of Medicinal Plant Development (IMPLAD), Chinese Academy of Medical Sciences & Peking Union Medical College (Key Laboratory of Bioactive Substances and Resources Utilization of Chinese Herbal Medicine, Ministry of Education & National Engineering Laboratory for Breeding of Endangered Medicinal Materials), Beijing, 100193 China

**Keywords:** Genetics, Plant sciences

## Abstract

*Radix Bupleuri* (Chaihu in Chinese) is a traditional Chinese medicine commonly used to treat colds and fevers. The root metabolome and transcriptome of two cultivars of *B. chinense* (BCYC and BCZC) and one of *B. scorzonerifolium* (BSHC) were determined and analyzed. Compared with BSHC, 135 and 194 differential metabolites were identified in BCYC and BCZC, respectively, which were mainly fatty acyls, organooxygen metabolites. A total of 163 differential metabolites were obtained between BCYC and BCZC, including phenolic acids and lipids. Compared with BSHC, 6557 and 5621 differential expression genes (DEGs) were found in BCYC and BSHC, respectively, which were annotated into biosynthesis of unsaturated fatty acid and fatty acid metabolism. A total of 4,880 DEGs existed between the two cultivars of *B. chinense*. The abundance of flavonoids in *B. scorzonerifolium* was higher than that of *B. chinense*, with the latter having higher saikosaponin A and saikosaponin D than the former. Pinobanksin was the most major flavonoid which differ between the two cultivars of *B. chinense*. The expression of *chalcone synthase* gene was dramatically differential, which had a positive correlation with the biosynthesis of pinobanksin. The present study laid a foundation for further research on biosynthesis of flavonoids and terpenoids of *Bupleurum* L.

## Introduction

*Radix Bupleuri* (thorowax root, Chaihu in Chinese) originates from the dried roots of perennial herbs from genus of *Bupleurum.* It has a long history of medicinal use in Asian countries such as China, Japan, and Korea. Pharmacological research has verified that Chaihu has diverse functions, including hepatoprotective, antitumor, antipyretic, analgesic, antibacterial, antiviral, anti-inflammatory, antioxidation, antidepression, antiepilepsy, and immune regulation^1–6^. It is commonly used to treat cold, fever, irregular menstruation, liver cancer, breast cancer, gastric ulcer, epilepsy, virus infection, and other diseases. It also regulates the central nervous system, cardiovascular system, digestive system, and immune system^7,8^. The antidepressant effect of Chaihu and traditional prescriptions with Chaihu as the principal component has been investigated. Clinical case studies have shown that Chaihu can alleviate functional dyspepsia symptoms and relieve depression and anxiety^9,10^.

Phytochemical studies have shown that Chaihu contains abundant natural metabolites, such as phenolics, lignans, flavonoids, terpenoids (triterpenoids and sterols), mono- and sesquiterpenes (essential oils), and polyacetylenes, which have anti-inflammatory, anticancer, and antidepression properties^11^. Saikosaponins (SSs) are a type of pentacyclic triterpenoid saponin and are generally considered as the main medicinal metabolites of Chaihu. Over 100 SS monomers have been isolated and divided into 14 types according to their different aglycones^12,13^. Among the extracted saponins, saikosaponin A (SSa), C (SSc), and D (SSd) represent the majority content of SSs^14^. Different SS monomers possess distinguishing pharmacological actions. SSa and SSd exert inhibitory effects on hepatoma, breast cancer, pancreatic cancer, and depression^15–19^. It was demonstrated that SSc may ameliorate renal damage and fibrosis in the accelerated nephrotoxic serum nephritis model^20^. The flavonoids in *Bupleurum chinense* DC. have antioxidant, bacteriostatic, and hepatoprotective functions^7^. The polysaccharides in *B. chinense* can enhance immunity and lower blood lipids^21^. Moreover, the volatile oils in Chaihu have antipyretic and analgesic effects.

In China, both species of *B. chinense* and *B. scorzonerifolium* Willd*.* have been officially designated as the source of Chaihu^22^, which are commonly called “*Bei-Chaihu*” and “*Nan-Chaihu*”, respectively. The SSs content of *Bei-Chaihu* was reported to be significantly higher than that of other varieties in the same genus, including *Nan-Chaihu.* The content of total flavonoids in *Bei-Chaihu* was lower than that in *Nan-Chaihu*^23^. The content of volatile oil was higher in *Nan-Chaihu* than in *Bei-Chaihu*^24^. *Bei-Chaihu* is generally considered to have stronger anti-inflammatory and hepatoprotective effects than *Nan-Chaihu*, with the latter having a stronger antipyretic effect than the former. *Bei-Chaihu* is dominantly circulated in the market due to the strong environmental adaptability and extensive plantation of *B. chinense*. It is primarily produced in Shanxi, Gansu, Hebei, and Henan. The cultivation of *B. scorzonerifolium* is limited to Heilongjiang province. Previous comparisons were mainly conducted between the two medicinal materials seldomly considering the influence of different planting environments. In transcriptome studies, genes involved in the biosynthetic pathway of SSs were identified in *B. chinense* and *B. scorzonerifolium*^25,26^*.* However*,* studies on the biosynthesis of flavonoids were seldom in Chaihu.

Metabolome and transcriptome association analysis can realize the coexpression analysis of genes involved in pathways of differentially biosynthesized metabolites. Functional annotation and metabolic-pathway enrichment analysis can also be combined to systematically analyze the correlation between plant physiological regulatory mechanisms^27^. In the present study, three sources of *Bupleurum* germplasm were used including two cultivars of *B. chinense* and one of *B. scorzonerifolium* which were simultaneously sown and collected. Their root metabolome and transcriptome were determined and analyzed. The difference between the two species of *B. chinense* and *B. scorzonerifolium* as well as the two cultivars of *B. chinense* were discussed. Considering SSs and flavonoids as the medicinal metabolites of Chaihu, and their potential differences which lied in *B. chinense* and *B. scorzonerifolium*, terpenoids and flavonoids were focused on. The result can lay a foundation for the deep mechanism exploration under the elaborately distinguished traditional usage of *Bei-Chaihu* and *Nan-Chaihu* which sourced from *B. chinense* and *B. scorzonerifolium*, respectively. Furthermore, the data obtained will be a great contribution for cloning functional genes related to the biosynthesis of medicinal components of *Bupleurum* L.

## Results

### Metabolomic profiling

**Figure 1:**
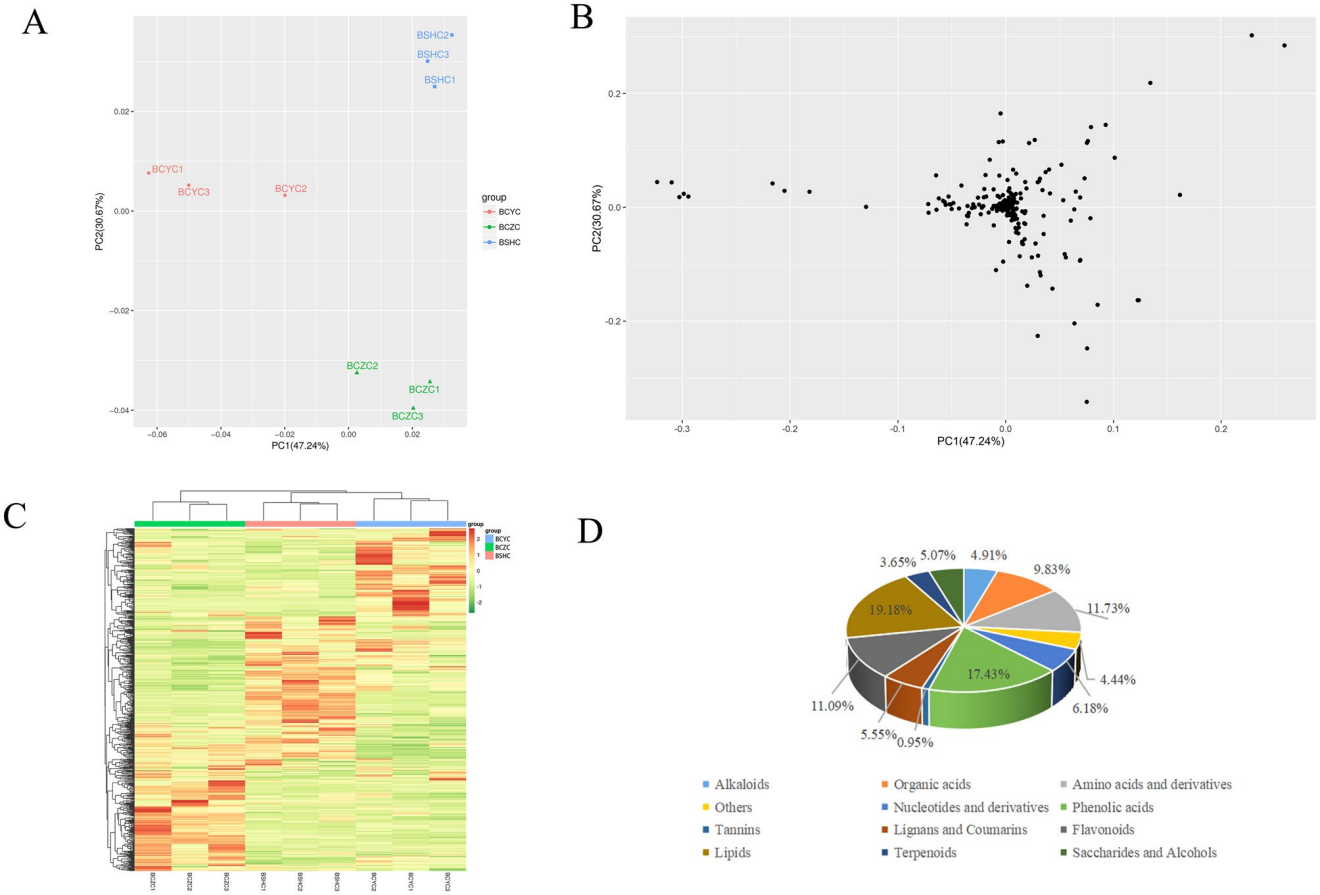
Systematic analysis of metabolome data of three *Bupleurum* cultivars (BCYC, BCZC, and BSHC). (**A**) PCA score plot of data obtained from metabolomic study showing obvious differences among the three cultivars. (**B**) PCA loading plot of total 631 detected metabolites. (**C**) Correlation heatmaps of total 631 detected metabolites indicating different metabolic characters among the three cultivars. (**D**) Classification pie chart of total 631 detected metabolites.

The total ion current (TIC) visual examination of all samples revealed a strong instrumental analysis signal, a large peak capacity, and good retention-time reproducibility. The figures of TIC were shown in Additional File 1 and the m/z value, molecular formula, error ppm, MS/MS fragmentation and type of ESI used, the *P*-value and VIP scores were shown in Additional File 2. A total of 631 metabolites were detected in the three *Bupleurum* cultivars. To compare the compositions of metabolites in the three *Bupleurum* cultivars, datasets obtained from UPLC-MS/MS were subjected to PCA. Results showed that the three *Bupleurum* cultivars were separated in the PC1 × PC2 score plots. Indeed, PC1 and PC2 in ESI+ and ESI− modes (47.24% and 30.67%, respectively) were clearly separated among the BCYC, BCZC, and BSHC (representing the three cultivars with the former two belonging to the species of *B. chinense* and the last one belonging to the species of *B. scorzonerifolium*) (Fig. 1A). For these metabolites, PCA accurately grouped all samples into distinct clusters, which reflected the obvious metabolic differences among the three *Bupleurum* cultivars. The PCA loading plot of total 631 detected metabolites was shown in Fig. 1B. Heatmap analysis of metabolomic data showed that the 631 metabolites were significantly separated into three clusters corresponding with three *Bupleurum* cultivars (Fig. 1C), indicating that the three different root materials exhibited different metabolic characters.

Based on the component analysis of all detected 631 metabolites, lipids were the most abundant metabolites, accounting for 19.18% of the total, and phenolic acids were the second most abundant, accounting for 17.43%. Furthermore, the abundance of amino acids and derivatives, flavonoids, organic acids, and nucleotides and derivatives accounted for 11.73%, 11.09%, 9.83%, and 6.18%, respectively. The proportion of terpenoids was 3.65% (Fig. 1D).

### Analysis of differential metabolites between* B. chinense* and *B. scorzonerifolium*

**Figure 2:**
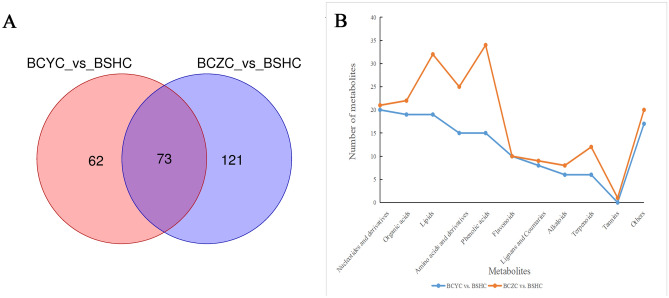
Differential metabolite analysis between *B. chinense* and *B.scorzonerifolium.* (**A**) Venn diagram of differential metabolites (BCYC vs. BSHC and BCZC vs. BSHC)*.* (**B**) Line chart of differential metabolites (BCYC vs. BSHC and BCZC vs. BSHC).

Among the 631 detected metabolites, 135 were found differentially accumulating between the control group BCYC and BSHC, including 35 down-regulated and 100 up-regulated metabolites, respectively. We also observed that 194 differentially accumulated metabolites were detected between the control group BCZC and BSHC, including 93 down-regulated and 101 up-regulated metabolites, respectively. Among these differential metabolites, 73 metabolites were identical between BCYC vs. BSHC and BCZC vs. BSHC (Fig. 2A). Among the 73metabolites, 57 were all higher in abundance in BSHC than BCYC and BCZC, including 14 nucleotides and derivatives, 10 lipids, 9 organic acids, 6 flavonoids, 5 amino acids and derivatives, 3 lignans and coumarins, 1 terpenoid, 1 phenolic acid, and 1 alkaloid. In addition, 12 differential metabolites were all lower in abundance in BSHC than in BCYC and BCZC, including 2 terpenoids. The remaining four metabolites were inconsistently up- and down-regulated between BCYC vs. BSHC and BCZC vs. BSHC. The large difference was found in metabolites between the two cultivars of *B. chinense* (BCYC and BCZC) (Fig. 2B). Phenolic acids varied most, followed by lipids, amino acids and derivatives, and terpenoids. Flavonoids and tannins varied slightly. In total, 15 phenolic acids were identified between BCYC and BSHC (3 up- and 12 down-regulated), 34 phenolic acids were identified between BCZC and BSHC (5 up- and 29 down-regulated). This indicates that phenolic acids differed greatly between the two cultivars of *B. chinense* (BCYC and BCZC), while the abundance of phenolic acids was higher in *B. chinense* than in *B. scorzonerifolium.* There were six terpenoids were identified between BCYC and BSHC (2 up- and 4 down-regulated), among which there were a total of four SSs, the abundance of three SSs was higher in BCZC than in BSHC. Moreover, 12 terpenoids were identified between BCZC and BSHC (3 up- and 9 down-regulated), of which there were a total of eight SSs, the abundance of six SSs was higher in BCZC than in BSHC. This indicates that there was a difference in the terpenoids between the two cultivars of *B. chinense*, the abundance of SSs was higher in *B. chinense* than in *B. scorzonerifolium.* A total of ten flavonoids were present between BCYC and BSHC (9 up- and 1 down-regulated), and ten flavonoids were present between BCZC and BSHC (7 up- and 3 down-regulated).

Ten flavonoids were differentially accumulating between BCYC and BSHC, and ten flavonoids between BCZC and BSHC. Among them, six flavonoids were identical between BCYC vs. BSHC and BCZC vs. BSHC, including 3-O-acetylpinobanksin, isorhamnetin-3-O-glucoside, isorhamnetin-7-O-glucoside (Brassicin), 6-hydroxykaempferol-7,6-O-diglucoside, isorhamnetin-3,7-O-diglucoside, and isorhamnetin-3-O-(6″-malonylglucoside)-7-O-glucoside. The abundance of the six flavonoids in BSHC were all higher than those in BCYC and BCZC. The largest difference in abundance was for 3-O-acetylpinobanksin (30,219.25- and 170.93-fold). This result indicated that the abundance of flavonoids in the *B. scorzonerifolium* was higher than that of *B. chinense.*

The abundance of six terpenoids was different between BCYC and BSHC, 12 terpenoids were different between BCZC and BSHC. Specifically, the abundance of 2α-hydroxyursolic acid was 63,354.94-fold higher in BSHC than in BCYC. Furthermore, 2α-hydroxyursolic acid was 9.89-fold higher in BSHC samples than in BCZC samples (Table 1). These results indicated significant metabolic differences between *B. chinense* and *B. scorzonerifolium.* There were four differentially accumulated SSs between BCYC and BSHC, including acetylsaikosaponin A, 16-keto-saikosaponin A, saikosaponin F, and acetylsaikosaponin F. Specifically, the acetylsaikosaponin A was 18.65-fold lower in BSHC than in BCYC. Except for saikosaponin F and acetylsaikosaponin F, other six differentially accumulated SSs were between BCZC and BSHC (Table 1). The abundance of SSa and SSd was higher in BCZC than in BSHC, the fold change (FC) were 3.51-fold and 2.74-fold, respectively. The results indicated that the abundance of SSa and SSd in *B. chinense* were all higher than those in *B. scorzonerifolium.*

**Table 1:** Comparison of differential metabolites (flavonoids and terpenoids) between *B. chinense* and *B. scorzonerifolium.* (Metabolites were bold which were identical between BCYC vs. BSHC and BCZC vs. BSHC; the parameters used to select differential compounds were FC > 1, *P*-value < 0.05 and VIP > 1).

Classification	BCYC vs. BSHC				BCZC vs. BSHC			
	Metabolites	FC	log2FC	regulated	Metabolites	FC	log2FC	regulated
Flavonoids	**3-O-Acetylpinobanksin**	**30,219.2540**	**14.8832**	**Up**	**3-O-Acetylpinobanksin**	**170.9301**	**7.4173**	**Up**
	**Isorhamnetin-3-O-Glucoside**	**13.1262**	**3.7144**	**Up**	**Isorhamnetin-3-O-Glucoside**	**7.9995**	**2.9999**	**Up**
	**Isorhamnetin-7-O-glucoside (Brassicin)**	**12.8224**	**3.6806**	**Up**	**Isorhamnetin-7-O-glucoside (Brassicin)**	**8.4943**	**3.0865**	**Up**
	**6-Hydroxykaempferol-7,6-O-Diglucoside**	**6.8209**	**2.7700**	**Up**	**6-Hydroxykaempferol-7,6-O-Diglucoside**	**5.9491**	**2.5727**	**Up**
	**Isorhamnetin-3,7-O-diglucoside**	**42.3666**	**5.4049**	**Up**	**Isorhamnetin-3,7-O-diglucoside**	**7.2311**	**2.8542**	**Up**
	**Isorhamnetin-3-O-(6″-malonylglucoside)-7-O-glucoside**	**18.6475**	**4.2209**	**Up**	**Isorhamnetin-3-O-(6″-malonylglucoside)-7-O-glucoside**	**10.4236**	**3.3818**	**Up**
	Kaempferol-4′-O-glucoside	2.1497	1.1041	Up	Isorhamnetin-3-O-arabinoside	3.4432	1.7837	Up
	Isorhamnetin-3-O-rutinoside-4'-O-glucoside	5.2885	2.4029	Up	Naringenin (5,7,4′-Trihydroxyflavanone)	0.1817	− 2.4603	Down
	Chrysoeriol-6,8-di-C-glucoside-4'-O-glucoside	5.2641	2.3962	Up	Quercetin-3-O-rhamnoside (Quercitrin)	0.0012	− 9.6467	Down
	Quercetin-3-O-(6″-galloyl) galactoside	0.0012	− 9.7275	Down	Quercetin-3-O-(6″-malonyl) glucoside	0.0018	− 9.1241	Down
Terpenoids	**2α-Hydroxyursolic acid**	**63,354.9360**	**15.9512**	**Up**	**2α-Hydroxyursolic acid**	**9.8872**	**3.3056**	**Up**
	**Saikosaponin F**	**0.4116**	**− 1.2808**	**Down**	**Saikosaponin F**	**0.2868**	**− 1.8017**	**Down**
	**AcetylSaikosaponin F**	**0.3244**	**− 1.6240**	**Down**	**AcetylSaikosaponin F**	**2,159.7356**	**11.0766**	**Up**
	**11-Keto-ursolic acid**	**0.0002**	**− 12.4532**	**Down**	**11-Keto-ursolic acid**	**0.0001**	**− 13.3737**	**Down**
	16-Keto-saikosaponin A	6.6990	2.7439	Up	Saikosaponin D	0.3638	− 1.4587	Down
	Acetylsaikosaponin A	0.0536	− 4.2211	Down	Saikosaponin A	0.2847	− 1.8124	Down
	–	–	–	–	Saikosaponin B4	0.4879	− 1.0352	Down
	–	–	–	–	Centellasaponin B	0.1527	− 2.7114	Down
	–	–	–	–	Saikosaponin I	0.1878	− 2.4130	Down
	–	–	–	–	AcetylSaikosaponin I	33.6037	5.0706	Up
	–	–	–	–	24,30-Dihydroxy-12(13)-enolupinol	0.0004	− 11.3474	Down
	–	–	–	–	Saikosaponin G	0.3392	− 1.5598	Down

### Analysis of intraspecific differential metabolites of *B. chinense*

The two cultivars of *B. chinense* whose seeds came from Shanxi and Hebei were chemically investigated. A total of 163 metabolites differed in abundance between the control group BCYC and BCZC, including 59 down-regulated and 104 up-regulated metabolites, respectively. These metabolites included 39 phenolic acids, 28 lipids, 25 amino acids and derivatives, and 12 organic acids. Eight lignans and coumarins were found between the BCYC and BCZC, accounting for 4.91%; eight alkaloids, accounting for 4.91%; seven flavonoids, accounting for 4.29%; and five SSs, accounting for 3.07%. This data correlated with Fig. 2B, which demonstrated the metabolic differences between the two cultivars of *B. chinense.* Among the seven analyzed flavonoids, the pinobanksin and naringenin were 336.10- and 7.86-fold higher, respectively, in BCZC than in BCYC (Table 2). Naringenin also existed in BCZC and BSHC as a differential metabolite. This indicated that naringenin not only existed between *B. chinense* and *B. scorzonerifolium*, but also existed between the two cultivars of *B. chinense*. By contrast, the abundance of the other five flavonoids were lower in BCZC than in BCYC, including quercetin-3-O-(2″-acetyl) glucuronide, isorhamnetin-3-O-(6″-acetylglucoside), chrysoeriol-7-O-(6″-malonyl) glucoside, isorhamnetin-3-O-(6″-malonylglucoside), and tamarixetin-3-O-(6″-malonyl) glucoside, which were no significant difference between *B. chinense* and *B. scorzonerifolium*.

**Table 2:** Intraspecific differential metabolites (flavonoids and terpenoids) from *B. chinense.* (BCYC vs. BCZC; The parameters used to select differential compounds were FC > 1, *P*-value < 0.05 and VIP > 1).

Classification	Metabolite	FC	log2FC	Regulated
Flavonoids	Pinobanksin	336.1034	8.3928	Up
	Naringenin (5,7,4′-Trihydroxyflavanone)	7.8678	2.9760	Up
	Quercetin-3-O-(2″-acetyl) glucuronide	0.1875	− 2.4149	Down
	Isorhamnetin-3-O-(6″-acetylglucoside)	0.2022	− 2.3065	Down
	Chrysoeriol-7-O-(6″-malonyl) glucoside	0.3730	− 1.4229	Down
	Isorhamnetin-3-O-(6″-malonylglucoside)	0.1829	− 2.4507	Down
	Tamarixetin-3-O-(6″-malonyl) glucoside	0.1745	− 2.5186	Down
Terpenoids	2α-Hydroxyursolic acid	6,407.8002	12.6456	Up
	Ursolic acid	1.6350	0.7093	Up
	Saikosaponin D	2.6528	1.4075	Up
	Saikosaponin G	2.3971	1.2613	Up
	Saikosaponin B4	1.8238	0.8670	Up
	Acetylsaikosaponin A	0.1960	− 2.3511	Down
	AcetylSaikosaponin F	0.0002	− 12.7006	Down

Seven terpenoids (5 up-regulated and 2 down-regulated) were identified to both comparisons in BCYC and BCZC are listed in Table 2. Specifically, the abundance of 2α-hydroxyursolic acid and ursolic acid was 6407.80- and 1.63- fold higher, respectively, in BCZC than in BCYC. The difference of 2α-hydroxyursolic acid between *B. chinense* and *B. scorzonerifolium* was also large. Furthermore, SSd was 2.65-fold higher in BCZC than in BCYC, which also was difference between BCZC and BSHC. This indicated that 2α-hydroxyursolic acid and SSd have differences not only between *B. chinense* and *B. scorzonerifolium*, but also between the two cultivars of *B. chinense*. Figures for the annotated MS/MS spectra of highly differentiating metabolites were shown in Additional File 3.

### Functional annotation and enrichment analysis of differential metabolites

**Figure 3:**
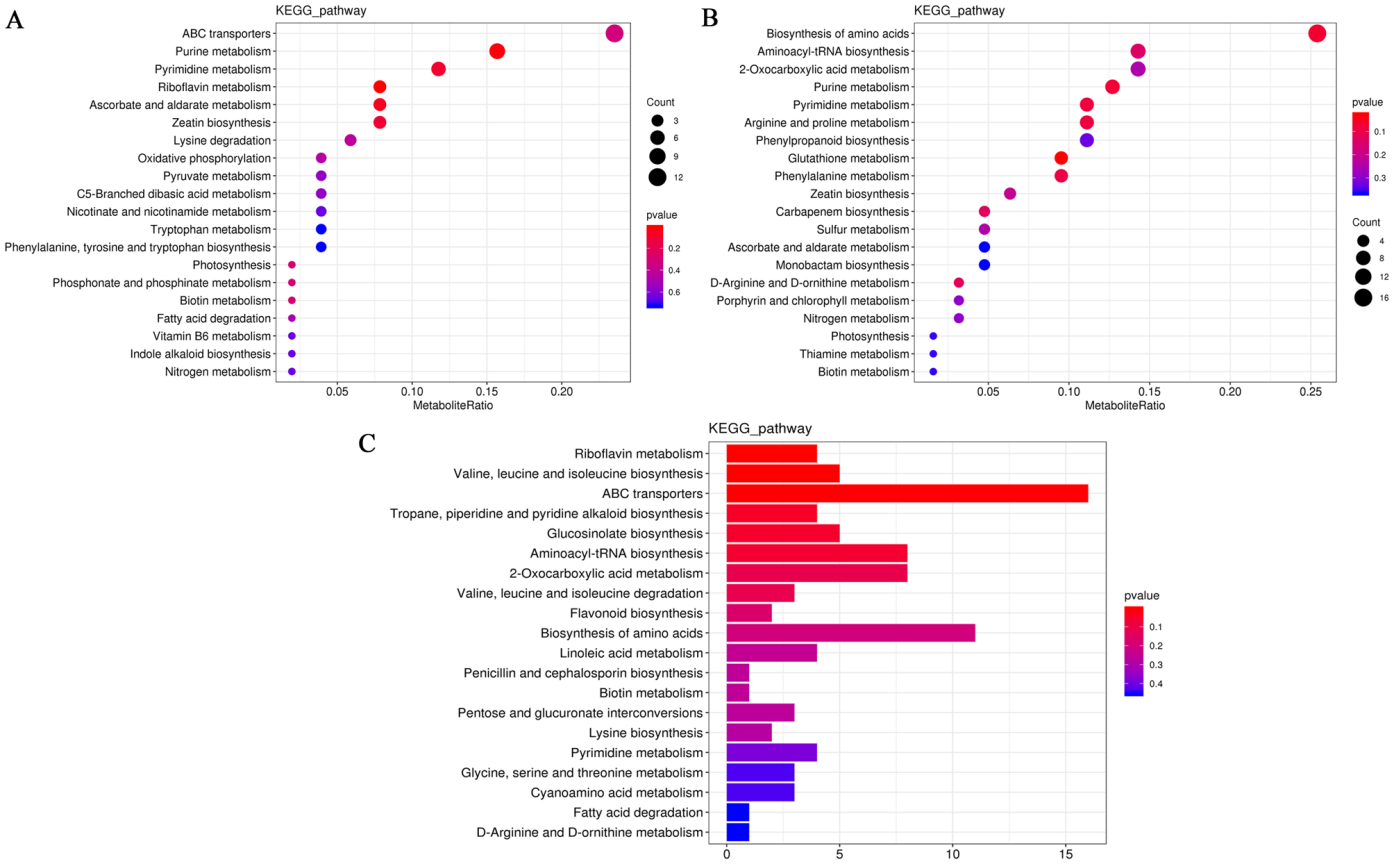
KEGG enrichment map of differential metabolites. The item of red dot is more significant than that of blue dot. (**A**) KEGG enrichment dotplot of differential metabolite (BCYC vs. BSHC). (**B**) KEGG enrichment dotplot of differential metabolite (BCZC vs. BSHC). (**C**) KEGG enrichment barplot of differential metabolite (BCYC vs. BCZC).

KEGG analysis is a crucial tool that can enable thorough understanding of specific metabolites’ synthesis processes, relevant gene functioning, and multiple genes interactions at the transcriptome level. Differential metabolites between BCYC and BSHC were mainly annotated into the ABC transporters, purine metabolism, pyrimidine metabolism, and riboflavin metabolism pathway (Fig. 3A). Differential metabolites between BCZC and BSHC were mainly annotated into the biosynthesis of amino acids, aminoacyl-tRNA biosynthesis, and 2-oxocarboxylic acid metabolism (Fig. 3B). Differential accumulated metabolites between the BCYC and BCZC were annotated into the KEGG pathway, including ABC transporters, biosynthesis of amino acids, aminoacyl-tRNA biosynthesis, and 2-oxocarboxylic acid metabolism pathway. In addition, the differential metabolites between BCYC and BCZC have been annotated into the flavonoid biosynthesis pathway (Fig. 3C)^28–30^.

### Assembly and annotation of Chaihu transcriptome

For further analysis, low-quality sequences were filtered out, and 62.94 Gb clean reads were obtained in the three *Bupleurum* cultivars. The clean data of each sample reached 6.43 Gb, and the percentage of Q30 bases was 92.54% or above, among which the average GC content was 43.56%. Using Stringtie software, the mapped reads were pieced together. We filtered out sequences that coded for peptides that were too short (< 50 amino acid residues) or contained only a single exon, and a total of 7922 new genes were discovered. BLAST software was used to perform sequence alignment with NR, Swiss-Prot, GO, COG, KOG, eggNOG, Pfam, and KEGG databases, and 7037 of new genes were functionally annotated. A total of 6993 unigenes with significant matches to the NR database were obtained, representing 99.37% of the total, the highest percentage.

### Identification and classification of DEGs

**Figure 4:**
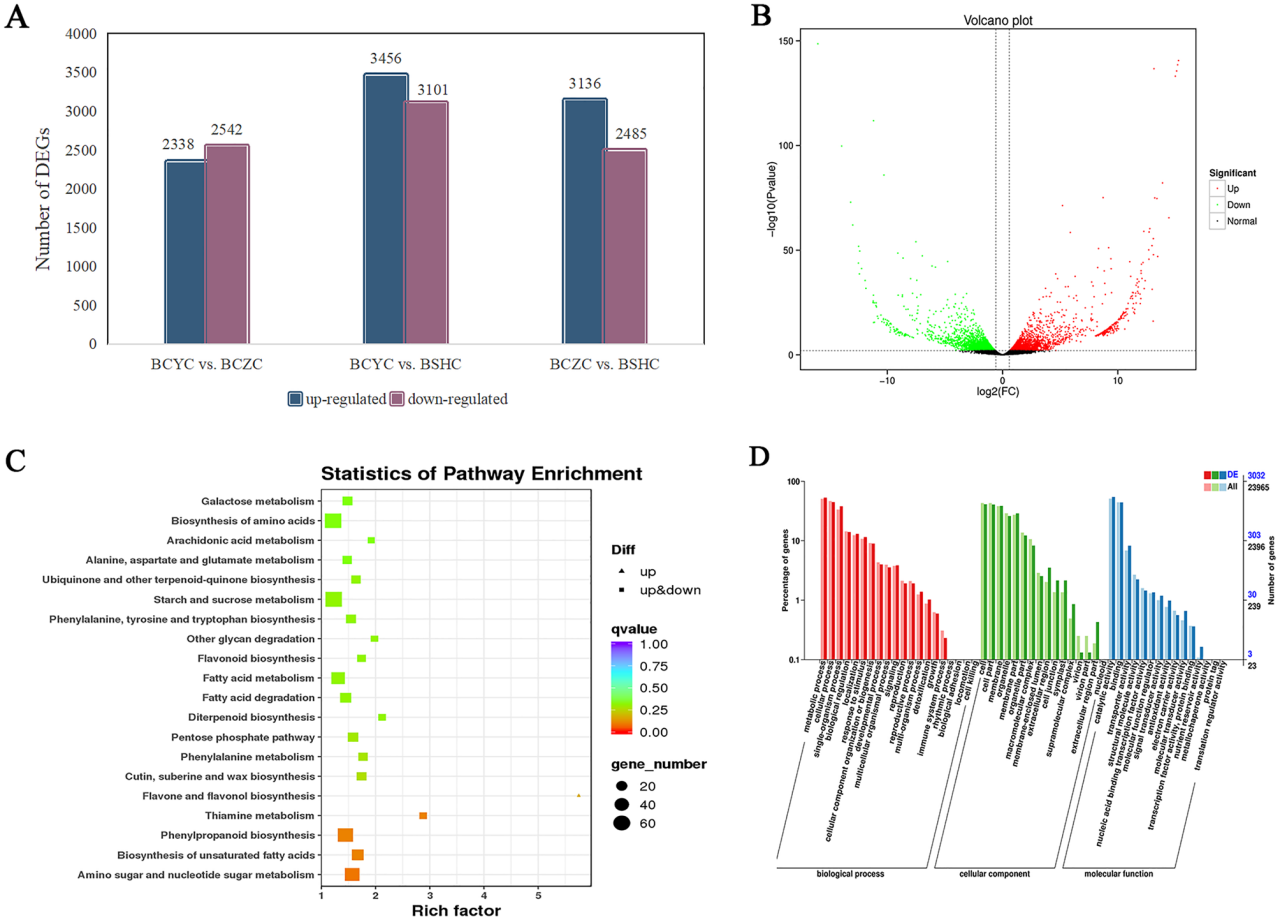
Correlation diagram of DEGs. (**A**) Statistical analysis of up- and down-regulation of DEGs in each group. (**B**) Volcano plot of DEGs (BCYC vs. BCZC). (**C**) Scatter plot of DEGs KEGG pathway enrichment (BCYC vs. BCZC). Each dot represents a KEGG pathway. The ordinate represents the name of the pathway, and the abscissa is the enrichment factor. A greater enrichment factor indicates a more significant enrichment level of DEGs in this pathway. The size of dots represents the number of DEGs enriched in the pathway. (**D**) GO enrichment analysis of DEGs (BCYC vs. BCZC).

The FPKM method was used to calculate unigene expression. A comparison of all up- and down-regulated genes according to the criteria of FC ≥ 1.5 at *P* < 0.01 revealed 4880 DEGs between BCYC and BCZC (2338 up- and 2542 down-regulated), 6557 DEGs between BCYC and BSHC (3456 up- and 3101 down-regulated), and 5621 DEGs between BCZC and BSHC (3136 up- and 2485 down-regulated) (Fig. 4A,B). Generally, more DEGs were in interspecies than intraspecies. The DEGs between BCYC and BCZC were mainly annotated into amino sugar and nucleotide sugar metabolism, phenylpropanoid biosynthesis, and biosynthesis of unsaturated fatty acids pathway. Furthermore, these DEGs between BCYC and BCZC were also annotated in the flavonoid biosynthesis pathways (Fig. 4C). The differential metabolites between BCYC and BCZC were also annotated with flavonoid biosynthesis. This demonstrated the consistence which lied in the differences of flavonoids abundance and related genes expression between the two cultivars of *B. chinense*. We functionally categorized the differentially expressed unigenes by using GO terms. A total of 3032 unigenes of DEGs were annotated between BCYC and BCZC. Genes in GO terms could be divided into three dominant categories: biological process, cellular component, and molecular function. The DEGs between BCYC and BCZC were the most abundant in the functional classification of cellular component category. The DEGs between BCYC and BCZC were primarily enriched in metabolic process in the biological process category. In the molecular function category, DEGs between BCYC and BCZC were significantly enriched in catalytic activity, binding, transporter activity, and structural molecule activity (Fig. 4D).

### Integrated analysis of the metabolome and transcriptome

**Figure 5:**
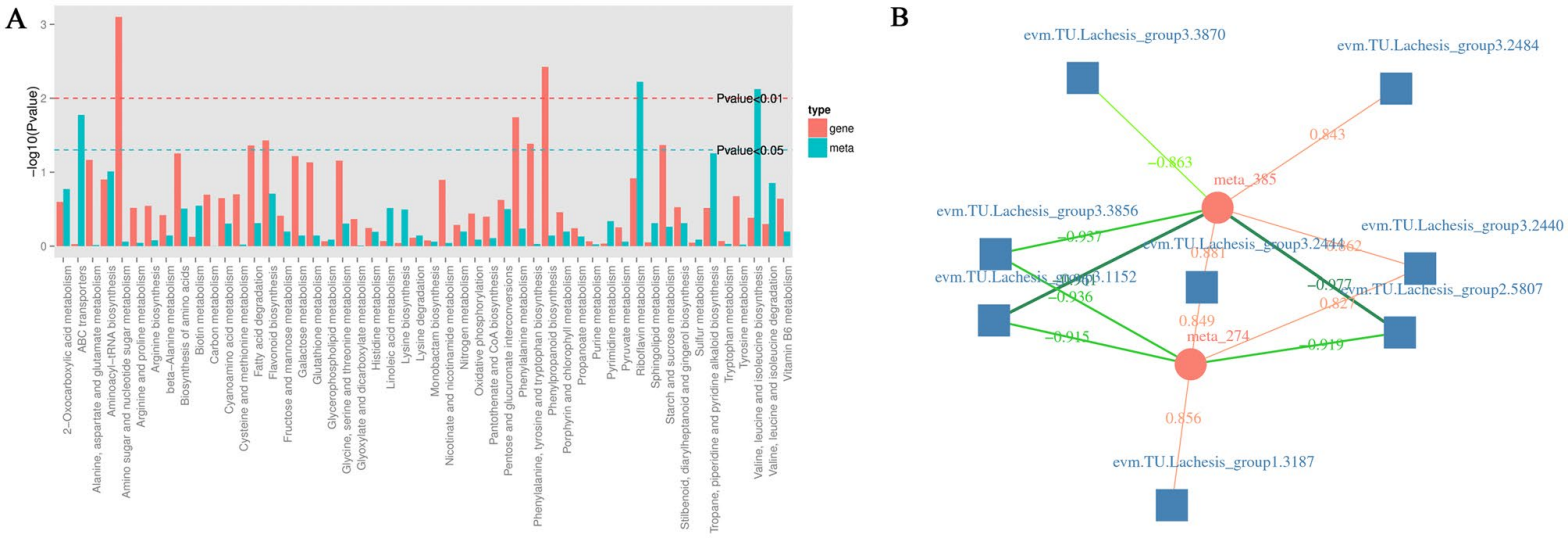
Combined metabolome and transcriptome correlation-analysis diagram. (**A**) Co-enrichment histogram of differential metabolites and DEGs (BCYC vs. BCZC). (**B**) Correlation network diagram. Circles represent metabolites, boxes represent genes, and values on the lines represent correlation coefficients, with positive correlation shown in red and negative correlation in green. A larger correlation coefficient corresponded with a wider line and darker color. meta_274: pinobanksin. *CHS*: evm.TU.L achesis_group1.3187; evm.TU.Lachesis_group3.2440; evm.TU.Lachesis_group3.2444. *CCoAOMT*: evm.TU. Lachesis_group3.1152. *HCT*: evm.TU. Lachesis_group2.5807. *CYP73A*: evm.TU. Lachesis_group3.3856.

An integrated analysis of metabolites and genes in the three *Bupleurum* germplasms revealed several common enriched pathways, including pyrimidine metabolism and biosynthesis of amino acids. Between BCYC and BCZC, differential metabolites and DEGs were enriched in 50 KEGG pathways (Fig. 5A). In the metabolome analysis between BCYC and BCZC, pinobanksin was the most significant detected flavonoid. In addition, the DEGs between BCYC and BCZC were also annotated in the flavonoid biosynthesis pathway in transcriptome analysis. The combined metabolomics and transcriptome analysis revealed a significant positive correlation between the chalcone synthase (CHS, EC: 2.3.1.74) and pinobanksin in the flavonoid biosynthesis pathway (ko00941). Furthermore, a negative correlation existed between the caffeoyl-CoA O-methyltransferase (CCoAOMT, EC 2.1.1.104) and pinobanksin, the correlation coefficient was − 0.92. Furthermore, shikimate O-hydroxycinnamoyltransferase (HCT, EC: 2.3.1.133) and trans-cinnamate 4-monooxygenase (CYP73A, EC: 1.14.14.91) were negatively correlated with pinobanksin, and the correlation coefficients were − 0.92 and − 0.94, respectively (Fig. 5B). Their regulatory relationships require further studies.

## Discussion

### Inter- and intra-species metabolic differences in Chaihu

Modern pharmacological research has shown that Chaihu has antipyretic, sedative, analgesic, antitussive, anti-inflammatory, antibacterial, antivirus, antitumor, antioxidation, antidepression, antiepilepsy, hepatoprotective, and choleretic effects. It has also regulating effects on the central nervous, cardiovascular, digestive, and immune systems. The difference in the types and contents of active ingredients between *B. chinense* and *B. scorzonerifolium* inevitably lead to differences in drug use. By our analysis, significant metabolite differences existed between the two species of *B. chinense* and *B. scorzonerifolium*, as well as within the intraspecific of *B. chinense*. Interspecies difference mainly includes nucleotides and derivatives, phenolic acids, lipids and organic acids, intraspecies difference mainly includes phenolic acids, lipids and amino acids and derivatives. The main medicinal components of Chaihu are flavonoids and terpenoids. The abundance of flavonoids in the *B. scorzonerifolium* was higher than that of *B. chinense*. In addition, the interspecific differences in the flavonoids of Chaihu were greater than the intraspecific differences. The abundance of SSa and SSd in *B. chinense* were all higher than those in *B. scorzonerifolium.* Our experimental material is the fresh roots of Chaihu, which is different from the dried roots and the roots under different concoction methods, but it also basically reflects the differences in metabolic synthesis between the two species of *B. chinense* and *B. scorzonerifolium*, which can provide a reference for the study of the two species to differentiate their medicinal use.

The differentially expressed flavonoids in this study mainly included isorhamnetin, quercetin, and pinobanksin. Some studies have shown that genes related to flavonoid biosynthesis include *CHS*, i*soflavone synthase (IFS)*, *flavanone 3-β-hydroxyalse (F3H)* and *dihydroflavonol 4-reductase (DFR)*^31–34^*.* In this study, the combined metabolomic and transcriptomic data revealed that *CHS* gene and the synthesis of the flavonoid pinobanksin were closely related. There is no report on *CHS* gene in Chaihu. The regulatory expression mechanism of *CHS* gene can be carried out subsequently, which can help to resolve the metabolism and regulation of flavonoid components in Chaihu.

The terpenoids differential metabolites in this study were all triterpenes, the main differential metabolite was SSs. With the in-depth study of molecular biology of medicinal plants, the biosynthetic pathway of SSs has been gradually clarified, in which the key enzyme genes regulating SSs have also been gradually explored, including *3-hydroxy-3-methylglutaryl-CoA reductase (HMGR)*, *isopentenyl diphosphate isomerase (IPPI), famesyl pryophosphate synthase (FPS)*, *squalene synthase (SS)*, *β-amyrin synthase (β-AS)*^35^. In this study, combined metabolomic and transcriptomic analyses showed no DEGs associated with triterpene between the two species of *B. chinense* and *B. scorzonerifolium*, as well as betweent the intraspecific of *B. chinense*.

In our study, we detected 163 differential metabolites between the two cultivars of *B. chinense*, including phenolic acids, lipids, amino acids and derivatives. These differential accumulated metabolites were annotated into the KEGG pathway, including ABC transporters, biosynthesis of amino acids, aminoacyl-tRNA biosynthesis, and 2-oxocarboxylic acid metabolism pathway. The expression levels of flavonoids and SSs were also significantly different between the two cultivars of *B. chinense.*

### The source of metabolic differences between *B. chinense* and *B. scorzonerifolium*

Factors affecting secondary metabolites are environmental conditions in addition to genetic diversity. Adversity stress causes an increase in the accumulation of secondary metabolites. Under drought stress, *B. chinense* indirectly increased the SSs content by regulating the gene expression of key enzymes of the SSs synthesis pathway^36^. Furthermore, stress can also affect the metabolism of flavonoids in *B. chinense*^37^. In this study, the seeds of the two species, *B. chinense* and *B. scorzonerifolium*, were sown in the same place and grown under the uniform management, thus basically excluding the influence of environmental conditions when compared with collecting and analyzing their roots which grew separately in their original places. It can be speculated that the differences we detected were mainly from genetic inheritance of both species. The combined metabolome and transcriptome of this study showed that there were differences in both primary and secondary metabolites between the two different species. Primary metabolic differences include nucleotides and amino acids, while secondary metabolites mainly include phenolic acids, lipids, organic acids, flavonoids, triterpenes, and alkaloids. The metabolic differences between *B. chinense* and *B. scorzonerifolium* can reflect the genetic diversity of Chaihu, which can further select and breed superior varieties for application to production.

### Flavonoids and related regulatory enzyme genes

Flavonoids are a class of secondary metabolites of polyphenols that widely exist in plants, and more than 10,000 flavonoids have been reported^38^. The flavonoids in *Bupleurum* species have hepatoprotective effects^39^. Pinobanksin is a flavonoid, which mainly comes from propolis. Pinobanksin could be considered as the promising xanthine oxidase inhibitors to prevent and treat hyperuricaemia or gout^40,41^. In addition, pinobanksin exerts anti-angiogenic effects by inhibiting endothelial cell apoptosis^42^. Pinobanksin can also act as an apoptosis inducer to induce apoptosis in lymphoma cells^43^. The biosynthetic pathway of flavonoids is ubiquitous in plants and produces extremely abundant secondary metabolites. There are a variety of key enzymes in flavonoid biosynthesis, including CHS. CHS is one of the key rate-limiting enzymes in plant flavonoid biosynthesis^44^. Studies have shown that CHS regulates stress-resistant flavonoids to help plants resist external stimuli, including ultraviolet radiation, mechanical damage, and nitrogen-deficient environments^45,46^. In addition, CHS involves in regulating a variety of plant stress responses and resisting pathogenic bacteria^47^. We combined metabolomics and transcriptome analysis revealed a significant positively correlation between the pinobanksin and *CHS* gene in the flavonoid biosynthesis pathway. The effect of *CHS* gene was likely to initiate the metabolism of downstream pathways. This finding provides support for the further study of flavonoids biosynthesis pathway.

## Materials and methods

### Plant materials

The three *Bupleurum* cultivars were two cultivars of *B. chinense* (named as BCYC and BCZC respectively) and one of *B. scorzonerifolium* (named as BSHC). The cultivars were identified by Prof. LIU Chunsheng from Beijing University of Chinese Medicine. The seeds of BCYC, BCZC, and BSHC, which were collected from the fields of Wanrong county of Shanxi province, She county of Hebei province, and Mingshui county of Heilongjiang province in China, respectively, were sown in the agricultural experiment field of our institutes in Beijing on April. After sowing, fully irrigation was conducted, and when the plant has four or five leaves, the redundant seedlings were removed to maintain the row spacing is about 20 cm × 30 cm. Then, during the whole growing season, only weeding manually was done. Until late of October, plants were at stage of fruit maturity and were about to come into winter dormancy. The plants with complete roots were dug out randomly and the above ground parts were cut off. Nine roots were collected from each of the three *Bupleurum* cultivars, and the roots from three plants of the same cultivar were mixed as one replicate when they were ground into powder. The freshly collected roots were immediately stored in liquid nitrogen, ground into powder using a mortar and pestle with liquid nitrogen, and stored at − 80 ℃ until RNA and metabolite extraction. Our experimental research and field studies on plants comply with relevant institutional, national, and international guidelines and legislation.

### Metabolite extraction and detection

Biological samples were freeze dried in a vacuum in a lyophilizer (Scientz-100F, Scientz, China). The freeze-dried root was crushed using a mixer mill (MM 400, Retsch, Germany) with a zirconia bead for 1.5 min at 30 Hz. Approximately 100 mg of powder was weighed and extracted overnight at 4 ℃ with 1.2 mL of 70% aqueous methanol^48^. 2-Chlorophenylalanine was used as internal standard. During overnight extraction, six times of vortex were conducted to improve the extraction efficiency. Following centrifugation at 12,000 rpm for 10 min, the extracts were absorbed (CNWBOND Carbon-GCB SPE Cartridge, 250 mg, 3 mL; Anpel, China) and filtered (SCAA-104; 0.22 µm pore size; Anpel, China) before ultraperformance liquid chromatography tandem mass spectrometry (UPLC-MS/MS) analysis^48^.

The samples extracts were injected onto a chromatographic column (Agilent SB-C18, 2.1 mm × 100 mm, 1.8 µm, Waters, USA) at a flow rate of 0.35 mL/min. The mobile phase consisted of solvent A (0.1% formic acid/water) and solvent B (0.1% formic acid/acetonitrile). Gradient grades of methanol and acetonitrile were purchased from Merck Company, Germany. Sample measurements were performed with a gradient program using the start conditions of 95% A and 5% B. Within 9 min, a linear gradient to 5% A and 95% B was programmed, and a composition of 5% A and 95% B was kept for 1 min. Subsequently, a composition of 95% A and 5% B was adjusted within 1.1 min and kept for 2.9 min. The column oven was set to 40 °C, and the injection volume was 4 µL. The effluent was alternatively connected to an ESI-triple quadrupole-linear ion trap (QTRAP)-MS. The data acquisition instrumentation system consists of Ultra Performance Liquid Chromatography (UPLC) (SHIMADZU Nexera X2, https://www.shimadzu.com.cn/) and Tandem mass spectrometry (MS/MS) (Applied Biosystems 4500 QTRAP, http://www.appliedbiosystems.com.cn/). The m/z range was 50–1250. Equal volumes of the different experimental samples were collected and blended as quality control samples and used to evaluate the stability of the results of the entire experimental process and the correlation analysis.

Both of ESI+ and ESI− modes were run, respectively. In one of both modes, stronger response was obtained for the same metabolite was chosen for further analysis. MS conditions mainly include: the ESI temperature was 550 °C; the MS voltage was 5500 V (positive mode)/− 4500 V (negative mode); the curtain gas (CUR) was 25 psi, and the collisional activated ionization (CAD) parameter was set to high. In the triple quadrupole (QQQ), each ion pair was scanned according to the optimized clustering voltage (DP) and collision energy (CE)^48^. Based on the self-built database BMK (biomarker database), the qualitative analysis of substances is carried out according to the secondary spectrum information. Isotopic signals, repeated signals containing K^+^, Na^+^ and NH4^+^, as well as repeated signals of fragment ions of other substances with higher molecular weight are removed during the analysis.

Metabolite quantification was completed by using the multiple reaction monitoring (MRM) mode of triple quadrupole MS. In MRM mode, the quadrupole firstly screened the precursor ions (parent ions) of the target material, and removed the corresponding ions of other molecular weight substances to preliminarily eliminate interference. After the precursor ions were induced and ionized by the collision chamber, they broke to form many fragment ions. The fragment ions were filtered by a triple quadrupole to select a required characteristic fragment ion, which eliminated the interference of non-target ions, making the quantification more accurate and more repeatable. After obtaining the MS analysis data of metabolites from different samples, the peak areas of all MS peaks were integrated, and the MS peaks of the same metabolite in different samples were integrated and corrected^49^.

### Metabolite data analysis

Metabolites were identified by comparing accurate masses, MS fragmentation patterns, and isotope patterns with the online metabolite databases of the Human Metabolome Database (http://www.hmdb.ca/). Our qualitative methods were in accordance with the methodological standards and was successfully applied^50,51^. For metabolites that do not have standards, they were compared with secondary spectra in public HMDB database. For the isomers, we marked them with “*” to represent that the two isomers cannot be distinguished (in Additional File 2). After obtaining the mass spectral analysis data of metabolites from different samples, the peak area of all mass-spectrum peaks was integrated, and integral correction of the mass-spectrum peaks of the same metabolite in different samples was performed^49^. Both unsupervised principal component analysis (PCA) and orthogonal projections to latent structure–discriminant analysis (OPLS-DA) were used to observe the overall differences in metabolic profiles between groups to identify their significant differential metabolites. The software used for PCA was Scales, Ggplot2, Ggrepel and Scatterplot3d; used for OPLS-DA was Ropls. The uv scaling method was used to perform multivariate analysis. Variable importance in projection (VIP) analysis was performed to evaluate the significance of metabolites. Fold change (FC) analysis using Ggplot2 software was used to evaluate differences of metabolites between compared groups. FC was the mean value of three replicates in the experimental group compared to the mean value of the three replicates in the control group. T-test was used to calculate the *P*-value which was used to determine the significance of the difference between the compared groups. The metabolites in each pair with VIP > 1, FC > 1 and *P*-value < 0.05 were considered as candidate metabolites with a significant difference. The metabolite sets were named as “A vs. B” to specify the comparing pair. "A" represents one cultivar as control group. “B” represents the other cultivar as corresponding treated group. The metabolites with a higher content in B than A are defined as up-regulated metabolites. The ones with lower content in B are defined as down-regulated metabolites. Identified metabolites were annotated using the Kyoto Encyclopedia of Genes and Genomes database (KEGG) compound database (http://www.kegg.jp/kegg/compound/), and annotated metabolites were then mapped to the KEGG Pathway Database (http://www.genome.jp/kegg/pathway.html). The annotation results of differential metabolite KEGG were enriched with ClusterProfiler^52^. Enrichplot and Ggplot2 were used to make KEGG enrichment plots, Pheatmap and RColorBrewer for cluster heat map analysis, and Pheatmap for correlation analysis.

### Transcriptome sequencing and analysis

Collected tissues were ground in liquid nitrogen and used for RNA extraction. Tiangen RNA prep Pure Plant Plus Kit plant (Tiangen, China) was applied for total RNA extraction. The extracted RNA was measured for concentration and purity by using Nanodrop (Thermo Fisher, USA). RNA integrity was measured with an Agilent 2100 (Agilent Technologies, USA). Library construction and RNA-seq were performed using Biomarker Technology Co. (Beijing, China). Illumina HiSeq 2500 platform (NEB, USA) was used for library preparation sequencing.

The raw reads were analyzed as follows. (1) The sequencing adapter and primer sequences in the reads were removed. (2) Low-quality data were filtered and reads with a ratio of N (N means that the base information cannot be determined) greater than 10% were removed. (3) Low-quality reads (the number of bases with a quality value Q ≤ 10 accounting for more than 50% of the total reads) were removed. Raw reads of sequencing were processed into clean reads by filtering low-quality reads. Reads were assembled using StringTie^53^. The data were normalized before analysis, that is, each metabolite of each sample divided by the total peak area of the sample. The method of fragments per kilobase of transcript per million fragments mapped (FPKM) was applied to calculate the expression levels of genes^54^. DESeq2 was used to identify differential expression genes (DEGs)^55^, where a negative binomial generalized linear model was adopted. Then, genes with FC ≥ 1.5 and *P*-value < 0.01 detected were assigned as differentially expressed. In the gene sets named as "A vs. B" to specify the comparing pair, "A" represents one cultivar as control group. "B" represents the other cultivar as corresponding treated group. The genes with a higher expression level in B than A are defined as up-regulated genes. The ones with lower expression level in B are defined as down-regulated genes. DEGs were annotated by the NR, Swiss-Prot, GO, COG, KOG, eggNOG, Pfam, and KEGG databases. Finally, GO and KEGG pathway-enrichment analyses were performed to reveal functional modules and signal pathways of interest.

### Integrated metabolome and transcriptome analyses

**Figure 6:**
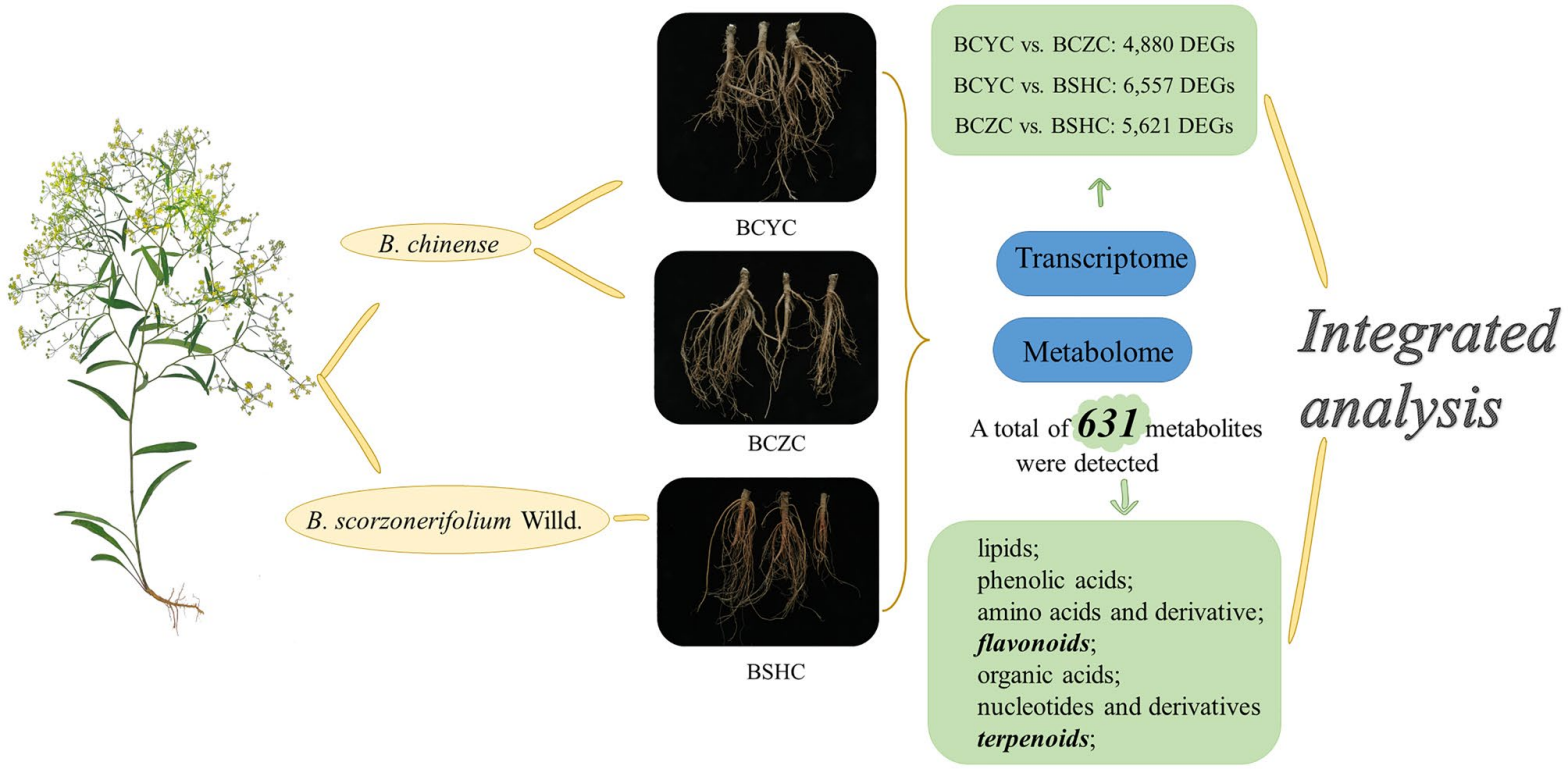
The figure of experimental materials and the experimental procedure.

Based on the metabolite content and gene expression data, Pearson correlation tests were used to detect associations between discriminant gene expression and discriminant metabolite content^56^. The mean of all biological replicates of three *Bupleurum* cultivars in the metabolome and transcriptome data were calculated. The coefficients were calculated with log_2_FC of each metabolite and log_2_FC of each DEG. Correlations between differential metabolites and DEGs were screened according to correlation coefficient (CC) and *P*-value. Only the detected associations with |CC| > 0.80 and *P*-value < 0.05 were selected. Metabolome and transcriptome relationships were visualized using Cytoscape (v3.8.2). Moreover, DEGs and differential metabolites were mapped to the KEGG pathway database to obtain their common pathway information. The experimental materials and the experimental procedure were shown in Fig. 6.

## Supplementary Information

Supplementary Information 1.

Supplementary Information 2.

Supplementary Information 3.

## Data Availability

The raw data of RNA-Seq has been deposited in the NCBI under the accession number PRJNA852406.
