# Supplementary figures and images for: Integrated metabolomics and transcriptomics analysis of roots of Bupleurum chinense and B. scorzonerifolium, two sources of medicinal Chaihu
Source: Sci Rep. 2022 Dec 26;12:22335. doi: 10.1038/s41598-022-27019-8 (PMC9792521; doi:10.1038/s41598-022-27019-8)

TIC of -MRM (722 pairs): from Sample 29 (A19051349a\_N) of MWBMK-19-065\_12\_JS4500-2\_C02\_MWDB4.0\_ZW\_20200730.wiff (Turbo Spra...

Max. 4.8e7 cps.

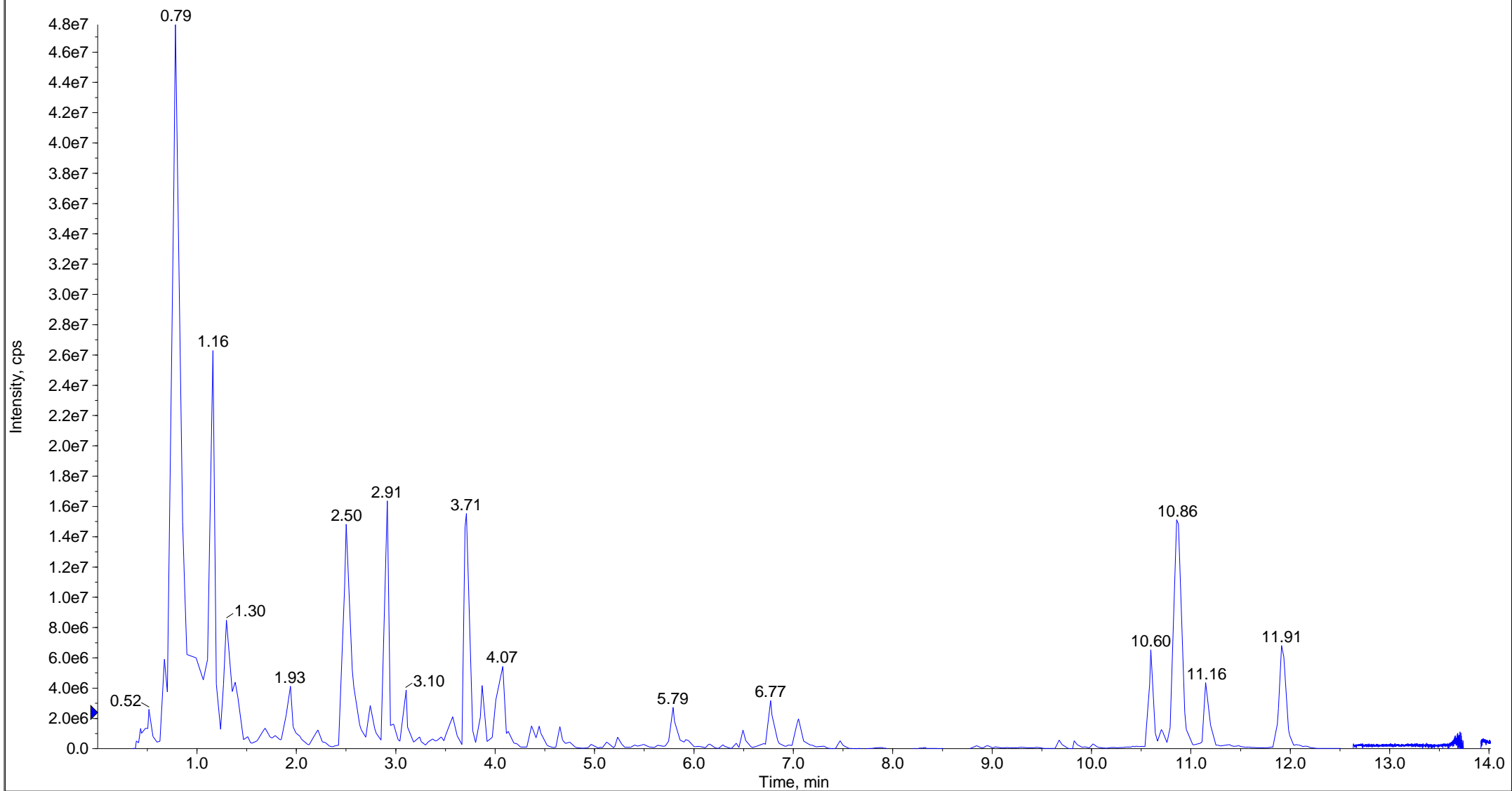

Supplement: Supplementary file 1 — Supplementary Information 1. [file 41598_2022_27019_MOESM1_ESM.zip › Additional file 1 The figure of total ion current/BCYC1-A19051349a_N.pdf]

■ TIC of +MRM (669 pairs): from Sample 11 (A19051349a\_P) of MWBMK-19-065\_12\_JS4500-2\_C02\_MWDB4.0\_ZW\_20200730.wiff (Turbo Spra...

Max. 5.3e7 cps.

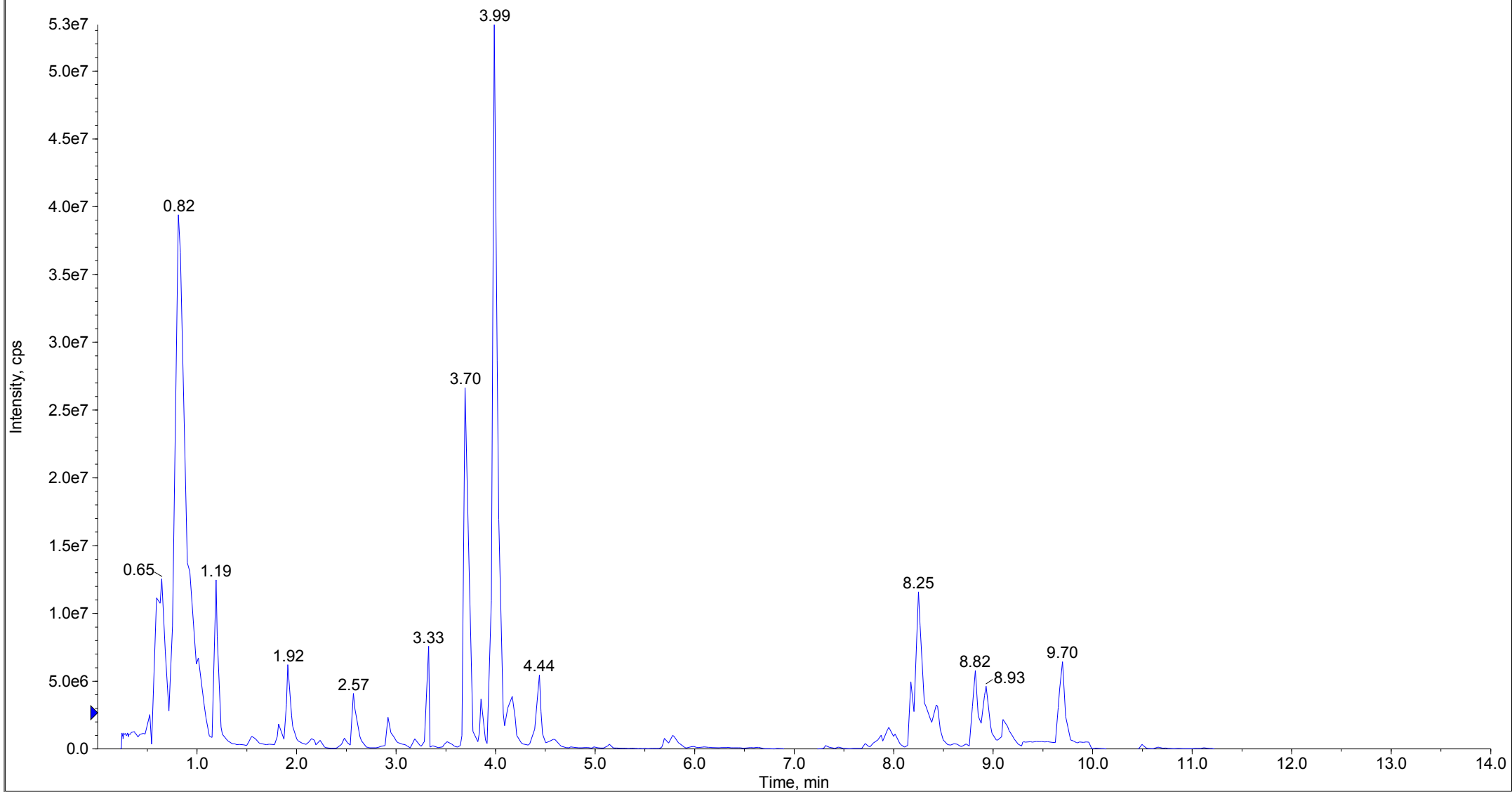

Supplement: Supplementary file 1 — Supplementary Information 1. [file 41598_2022_27019_MOESM1_ESM.zip › Additional file 1 The figure of total ion current/BCYC1-A19051349a_P.pdf]

TIC of -MRM (722 pairs): from Sample 30 (A19051350a\_N) of MWBMK-19-065\_12\_JS4500-2\_C02\_MWDB4.0\_ZW\_20200730.wiff (Turbo Spra...

Max. 4.3e7 cps.

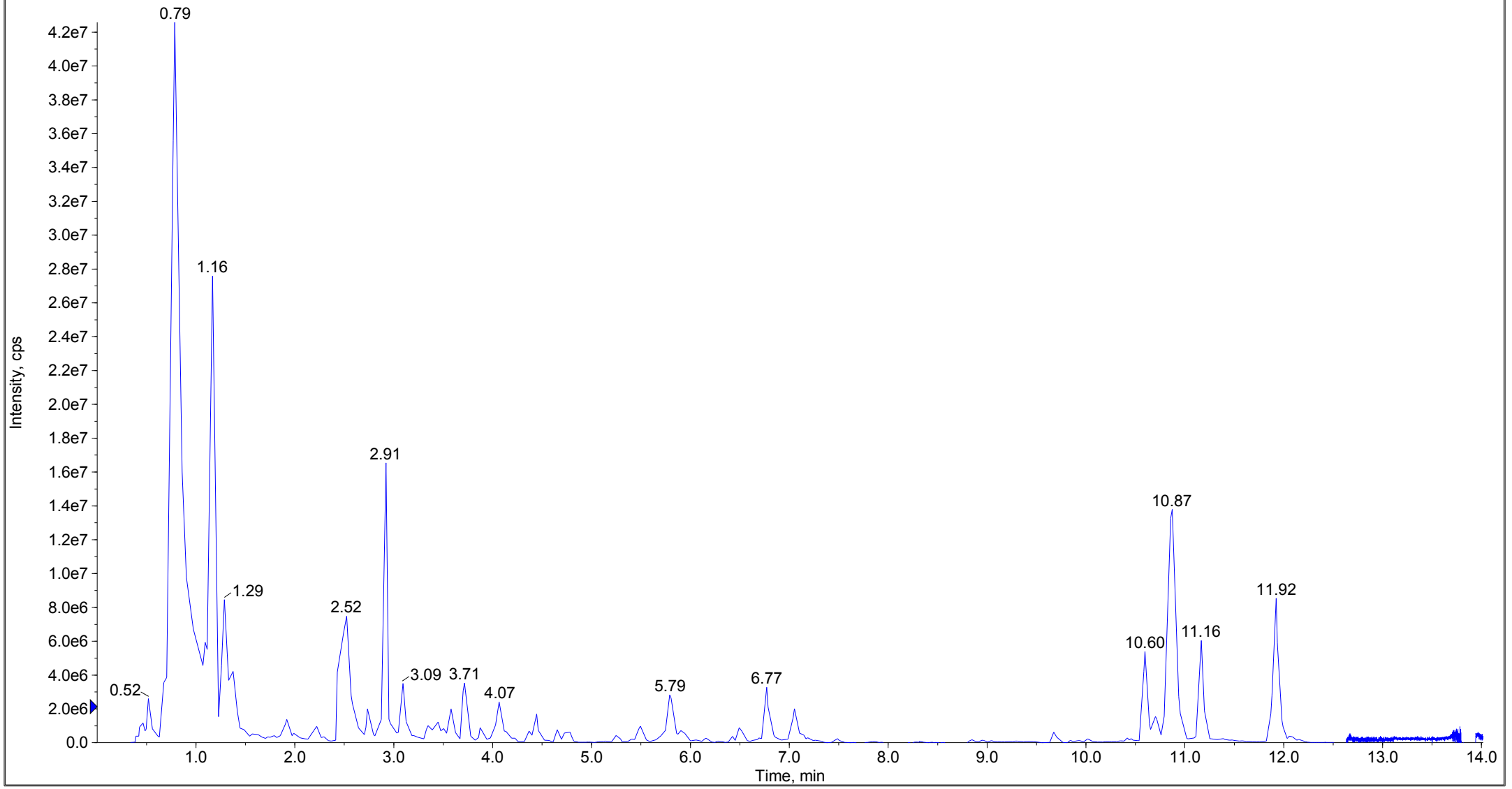

Supplement: Supplementary file 1 — Supplementary Information 1. [file 41598_2022_27019_MOESM1_ESM.zip › Additional file 1 The figure of total ion current/BCYC2-A19051350a_N.pdf]

TIC of +MRM (669 pairs): from Sample 12 (A19051350a\_P) of MWBMK-19-065\_12\_JS4500-2\_C02\_MWDB4.0\_ZW\_20200730.wiff (Turbo Spra...

Max. 3.0e7 cps.

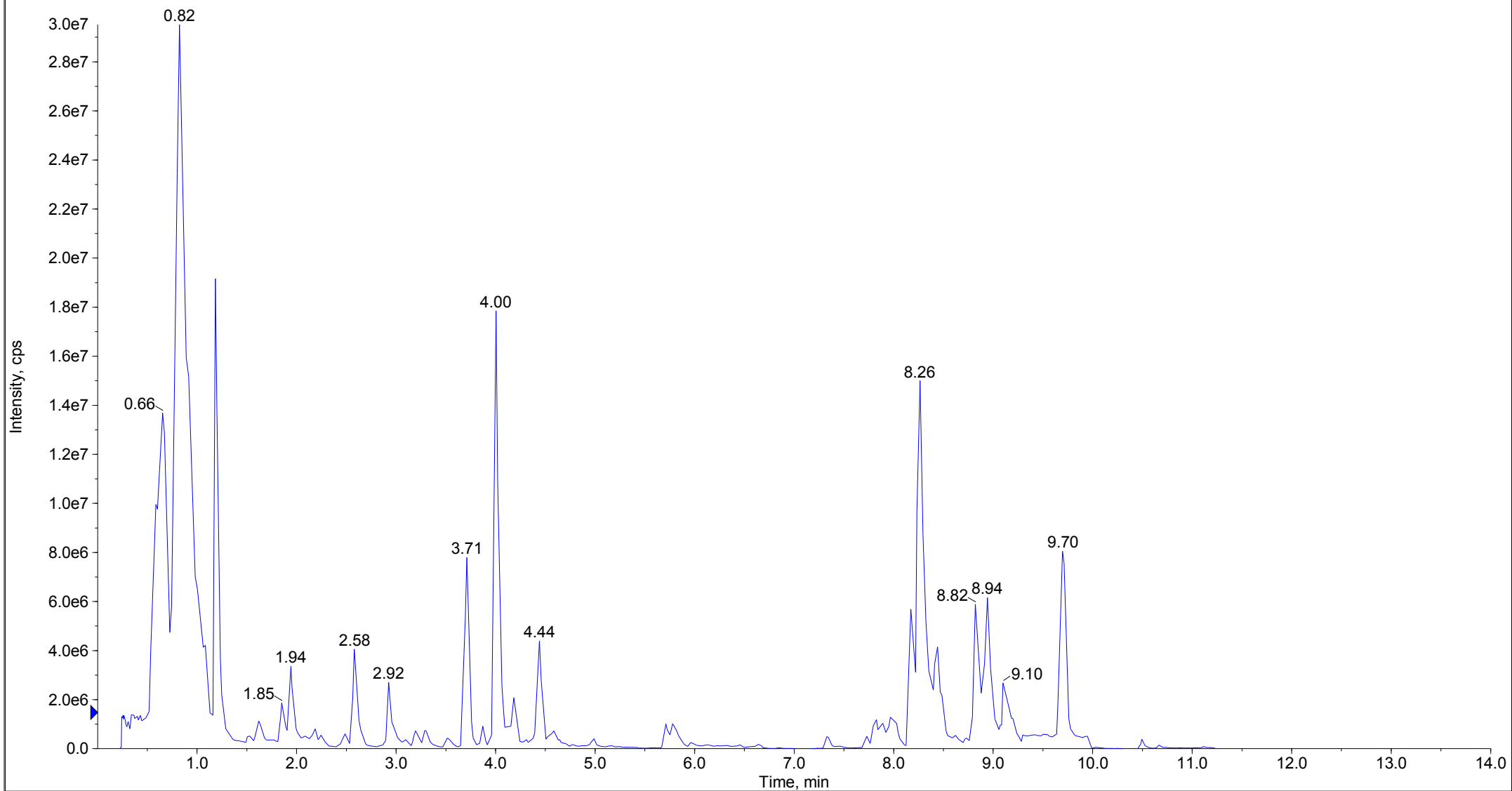

Supplement: Supplementary file 1 — Supplementary Information 1. [file 41598_2022_27019_MOESM1_ESM.zip › Additional file 1 The figure of total ion current/BCYC2-A19051350a_P.pdf]

TIC of -MRM (722 pairs): from Sample 31 (A19051351a\_N) of MWBMK-19-065\_12\_JS4500-2\_C02\_MWDB4.0\_ZW\_20200730.wiff (Turbo Spra...

Max. 4.2e7 cps.

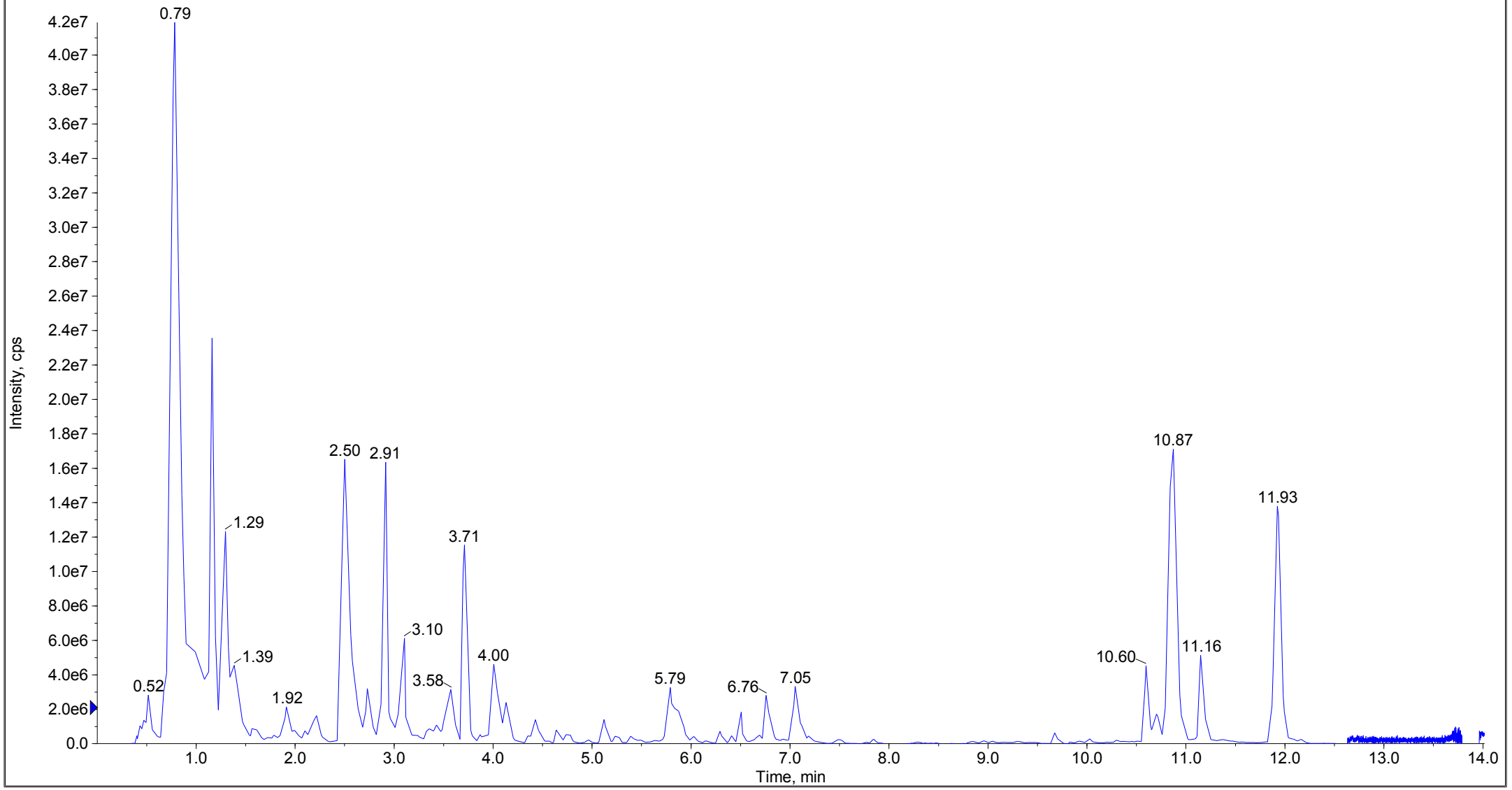

Supplement: Supplementary file 1 — Supplementary Information 1. [file 41598_2022_27019_MOESM1_ESM.zip › Additional file 1 The figure of total ion current/BCYC3-A19051351a_N.pdf]

TIC of +MRM (669 pairs): from Sample 13 (A19051351a\_P) of MWBMK-19-065\_12\_JS4500-2\_C02\_MWDB4.0\_ZW\_20200730.wiff (Turbo Spra...

Max. 6.4e7 cps.

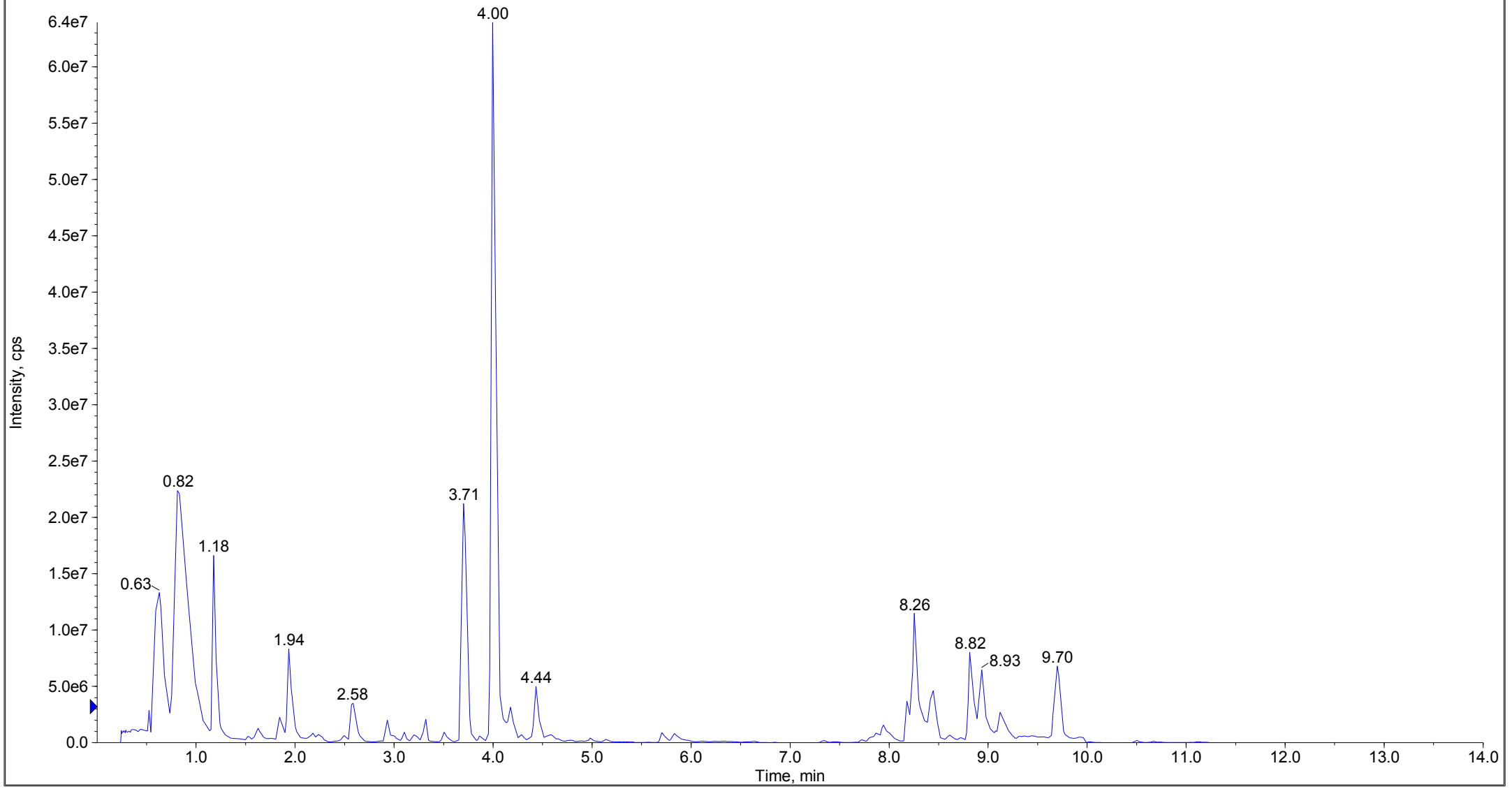

Supplement: Supplementary file 1 — Supplementary Information 1. [file 41598_2022_27019_MOESM1_ESM.zip › Additional file 1 The figure of total ion current/BCYC3-A19051351a_P.pdf]

TIC of -MRM (722 pairs): from Sample 24 (A19051340a\_N) of MWBMK-19-065\_12\_JS4500-2\_C02\_MWDB4.0\_ZW\_20200730.wiff (Turbo Spra...

Max. 4.1e7 cps.

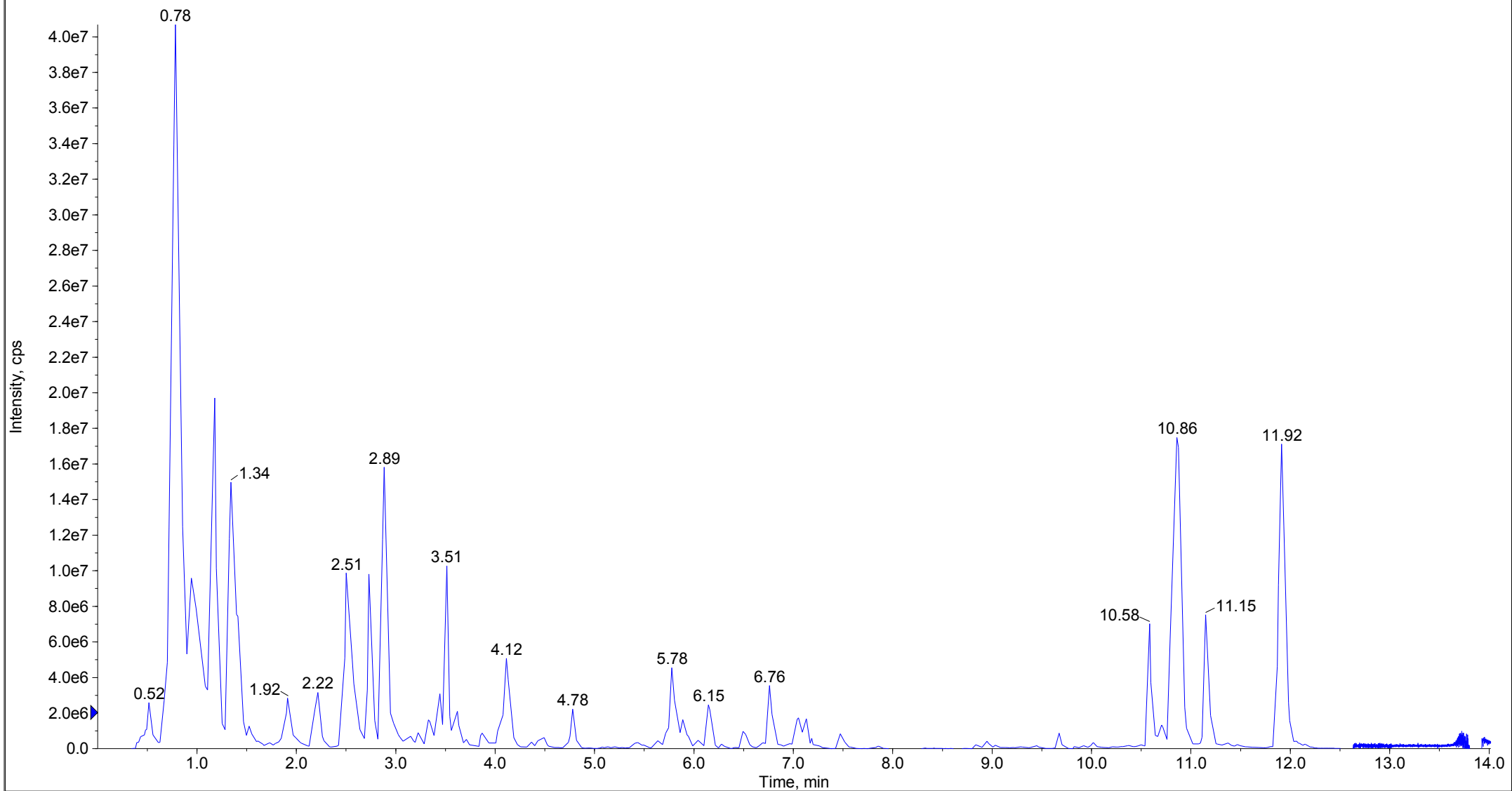

Supplement: Supplementary file 1 — Supplementary Information 1. [file 41598_2022_27019_MOESM1_ESM.zip › Additional file 1 The figure of total ion current/BCZC1-A19051340a_N.pdf]

■ TIC of +MRM (669 pairs): from Sample 6 (A19051340a\_P) of MWBMK-19-065\_12\_JS4500-2\_C02\_MWDB4.0\_ZW\_20200730.wiff (Turbo Spray...

Max. 2.8e7 cps.

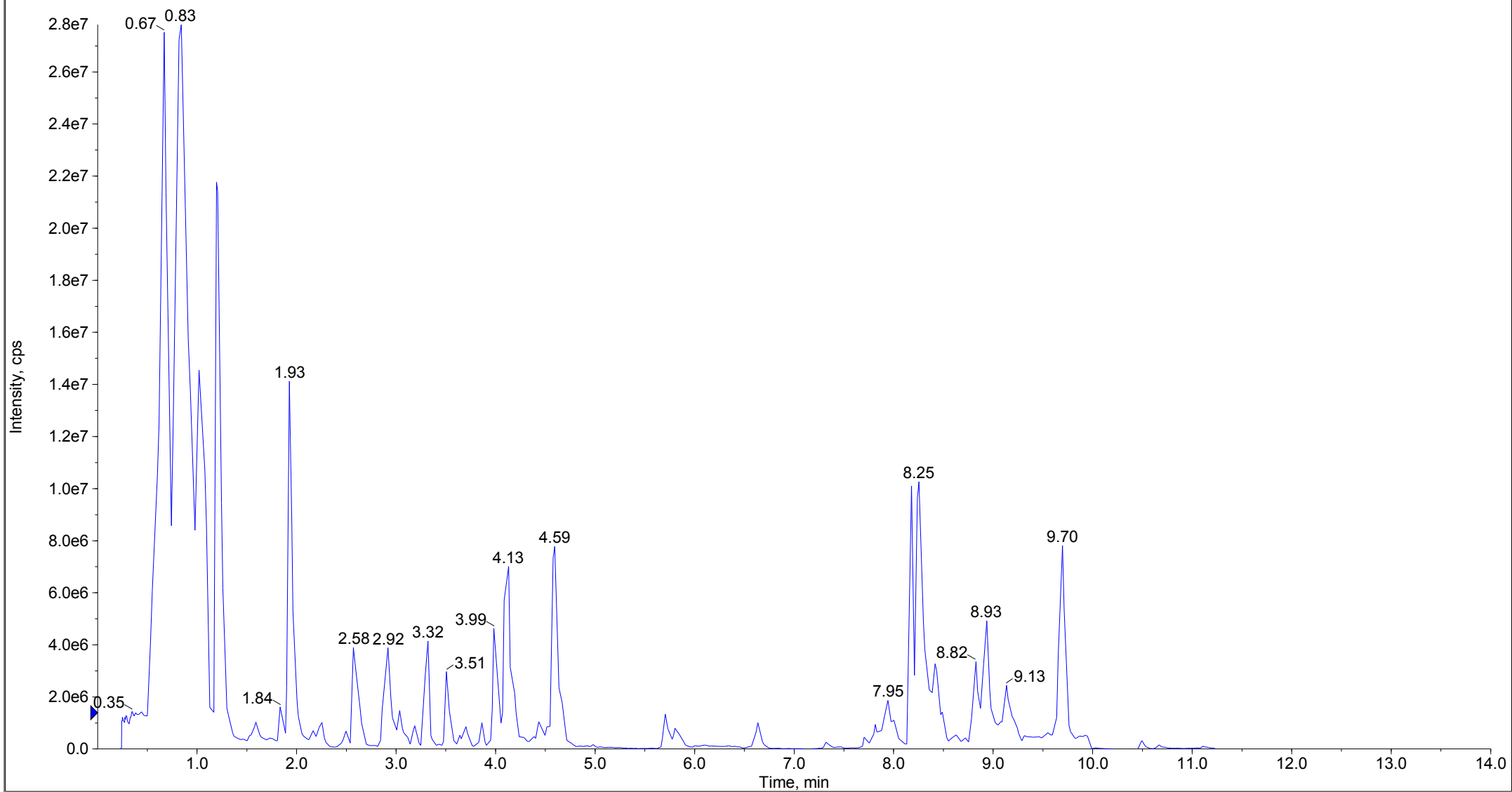

Supplement: Supplementary file 1 — Supplementary Information 1. [file 41598_2022_27019_MOESM1_ESM.zip › Additional file 1 The figure of total ion current/BCZC1-A19051340a_P.pdf]

TIC of -MRM (722 pairs): from Sample 25 (A19051343a\_N) of MWBMK-19-065\_12\_JS4500-2\_C02\_MWDB4.0\_ZW\_20200730.wiff (Turbo Spra...

Max. 3.8e7 cps.

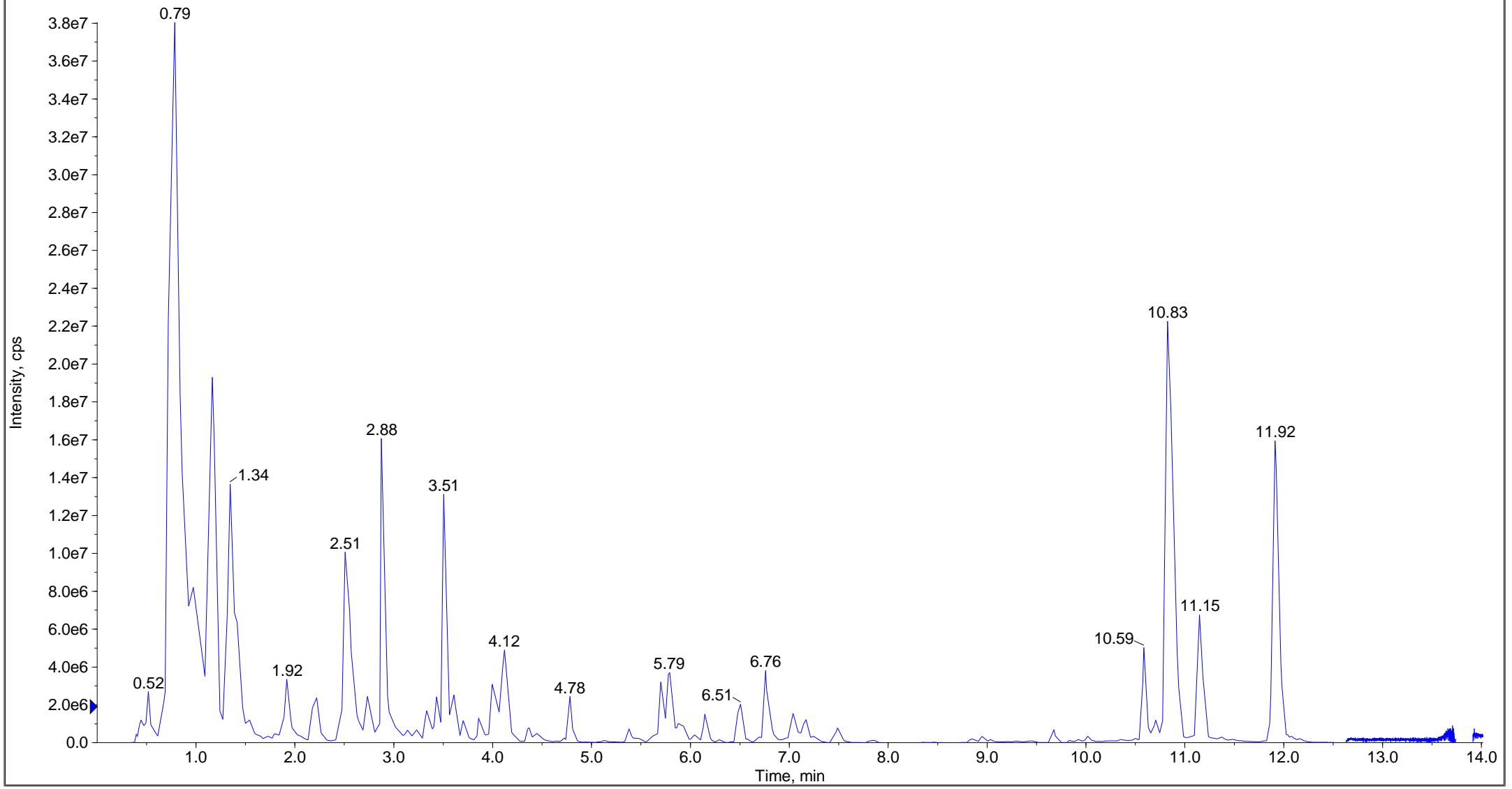

Supplement: Supplementary file 1 — Supplementary Information 1. [file 41598_2022_27019_MOESM1_ESM.zip › Additional file 1 The figure of total ion current/BCZC2-A19051343a_N.pdf]

TIC of +MRM (669 pairs): from Sample 7 (A19051343a\_P) of MWBMK-19-065\_12\_JS4500-2\_C02\_MWDB4.0\_ZW\_20200730.wiff (Turbo Spray...

Max. 5.8e7 cps.

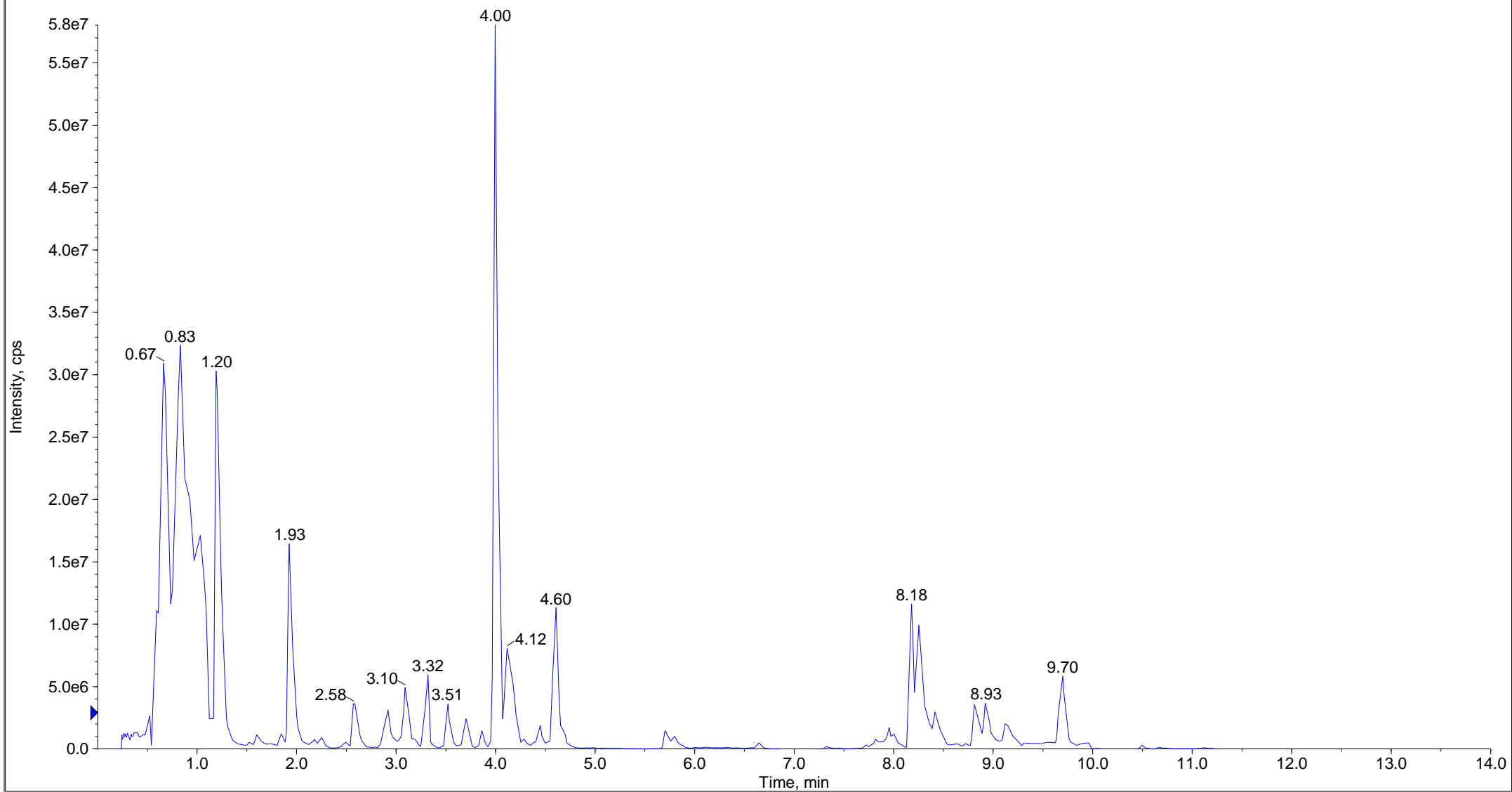

Supplement: Supplementary file 1 — Supplementary Information 1. [file 41598_2022_27019_MOESM1_ESM.zip › Additional file 1 The figure of total ion current/BCZC2-A19051343a_P.pdf]

TIC of -MRM (722 pairs): from Sample 26 (A19051346a\_N) of MWBMK-19-065\_12\_JS4500-2\_C02\_MWDB4.0\_ZW\_20200730.wiff (Turbo Spra...

Max. 4.1e7 cps.

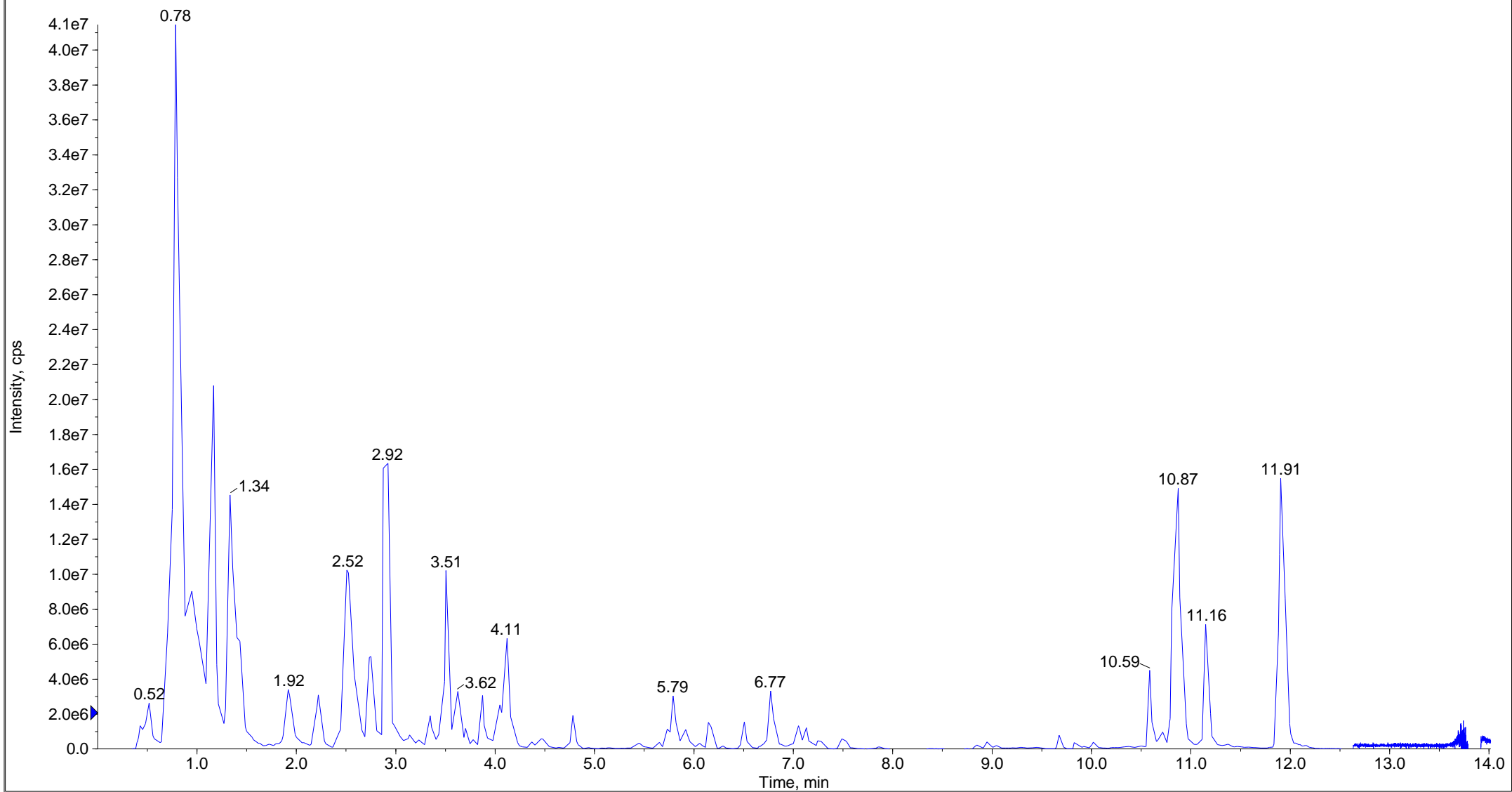

Supplement: Supplementary file 1 — Supplementary Information 1. [file 41598_2022_27019_MOESM1_ESM.zip › Additional file 1 The figure of total ion current/BCZC3-A19051346a_N.pdf]

TIC of +MRM (669 pairs): from Sample 8 (A19051346a\_P) of MWBMK-19-065\_12\_JS4500-2\_C02\_MWDB4.0\_ZW\_20200730.wiff (Turbo Spray...

Max. 3.8e7 cps.

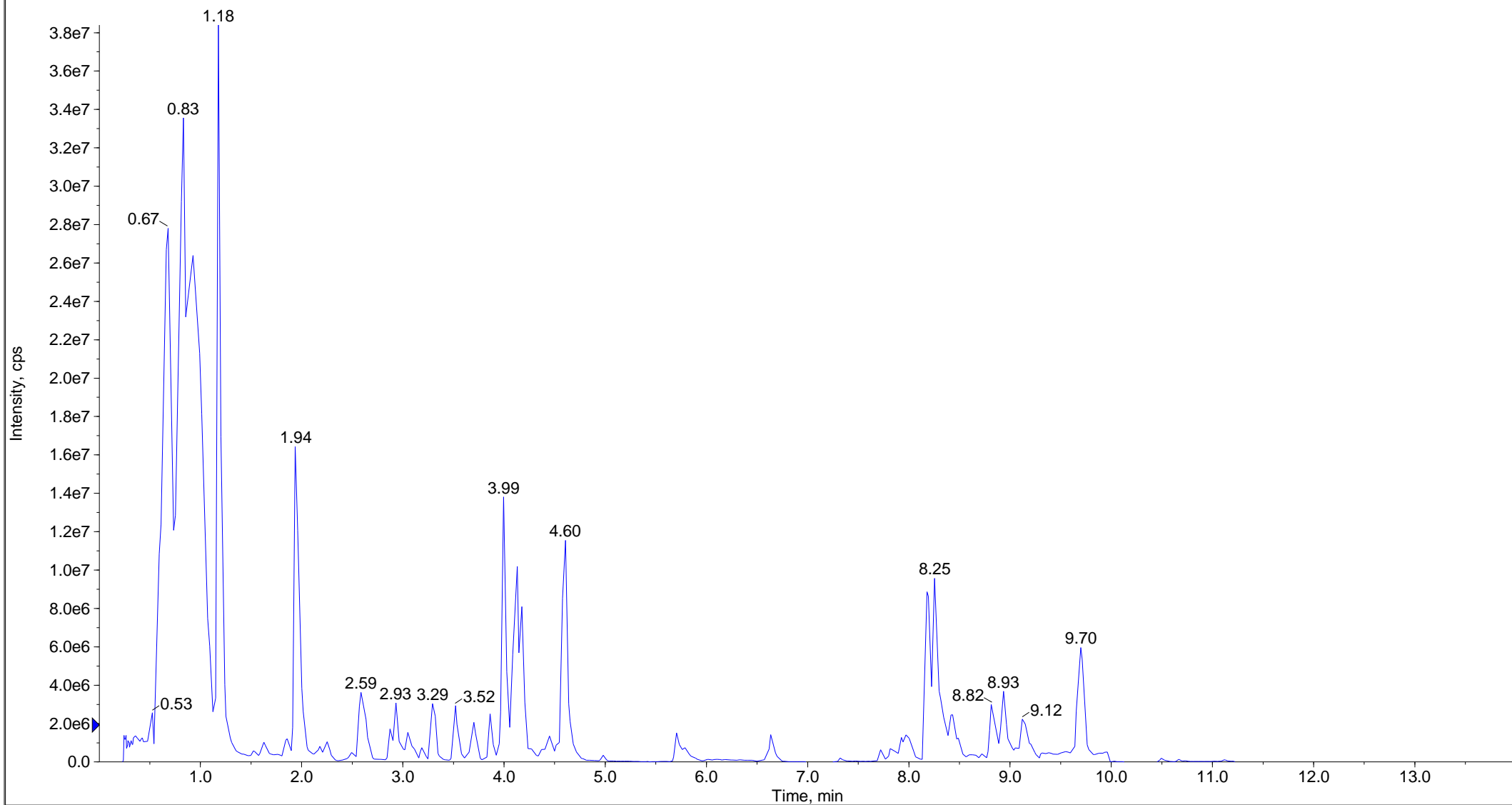

Supplement: Supplementary file 1 — Supplementary Information 1. [file 41598_2022_27019_MOESM1_ESM.zip › Additional file 1 The figure of total ion current/BCZC3-A19051346a_P.pdf]

TIC of -MRM (722 pairs): from Sample 21 (A19051331a\_N) of MWBMK-19-065\_12\_JS4500-2\_C02\_MWDB4.0\_ZW\_20200730.wiff (Turbo Spra...

Max. 4.0e7 cps.

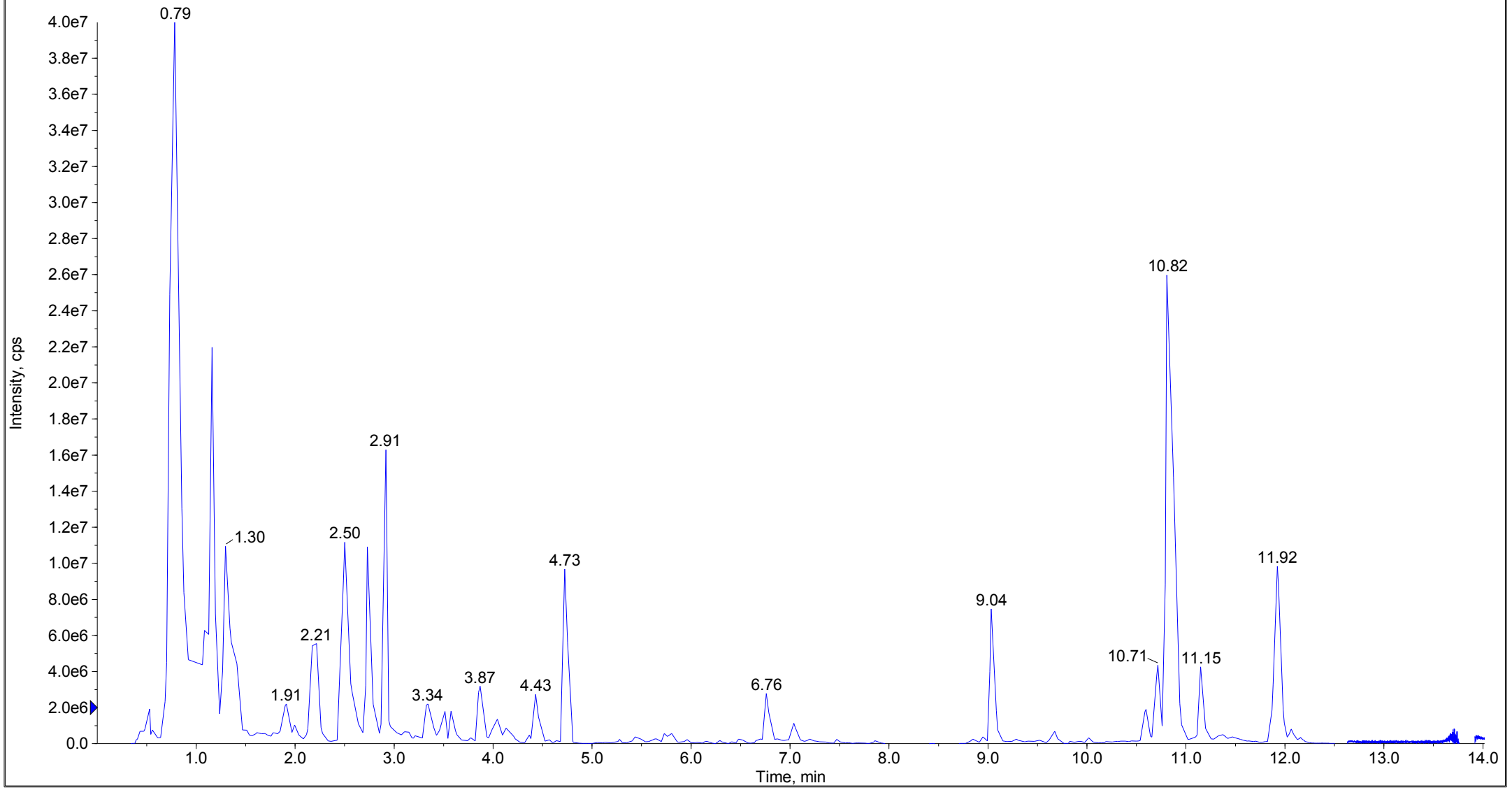

Supplement: Supplementary file 1 — Supplementary Information 1. [file 41598_2022_27019_MOESM1_ESM.zip › Additional file 1 The figure of total ion current/BSHC1-A19051331a_N.pdf]

TIC of +MRM (669 pairs): from Sample 3 (A19051331a\_P) of MWBMK-19-065\_12\_JS4500-2\_C02\_MWDB4.0\_ZW\_20200730.wiff (Turbo Spray...

Max. 3.3e7 cps.

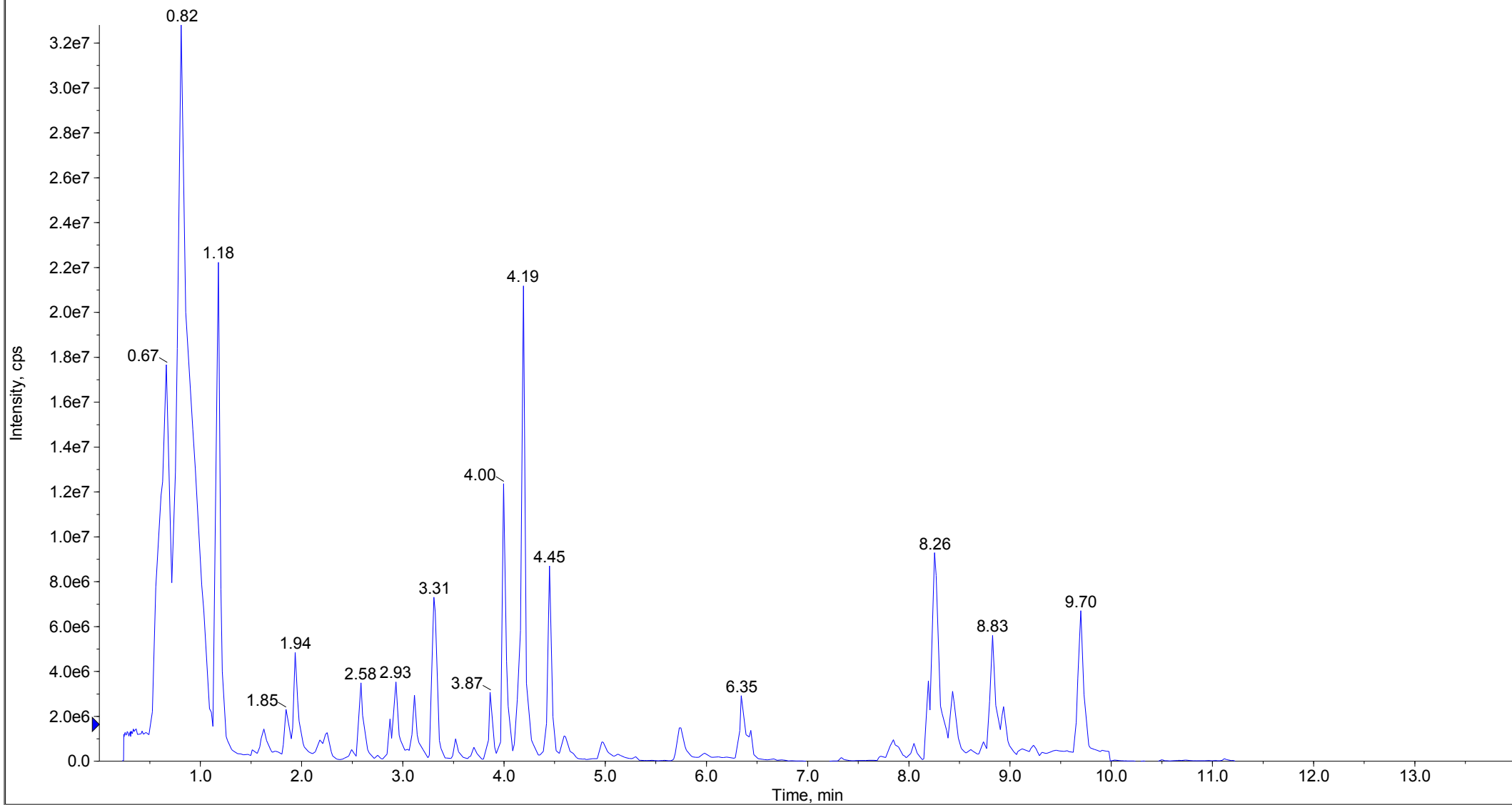

Supplement: Supplementary file 1 — Supplementary Information 1. [file 41598_2022_27019_MOESM1_ESM.zip › Additional file 1 The figure of total ion current/BSHC1-A19051331a_P.pdf]

TIC of -MRM (722 pairs): from Sample 22 (A19051334a\_N) of MWBMK-19-065\_12\_JS4500-2\_C02\_MWDB4.0\_ZW\_20200730.wiff (Turbo Spra...

Max. 3.9e7 cps.

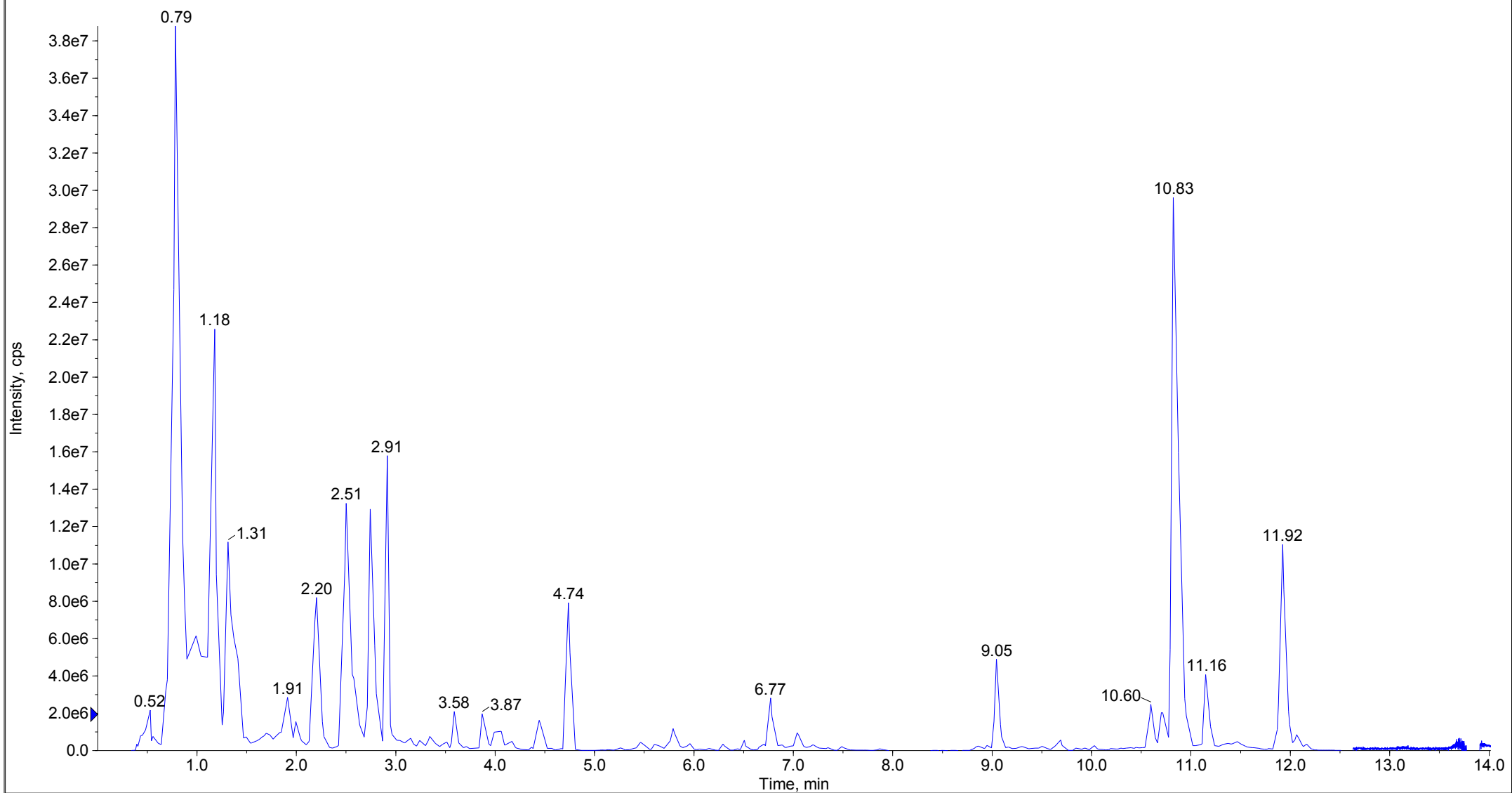

Supplement: Supplementary file 1 — Supplementary Information 1. [file 41598_2022_27019_MOESM1_ESM.zip › Additional file 1 The figure of total ion current/BSHC2-A19051334a_N.pdf]

TIC of +MRM (669 pairs): from Sample 4 (A19051334a\_P) of MWBMK-19-065\_12\_JS4500-2\_C02\_MWDB4.0\_ZW\_20200730.wiff (Turbo Spray...

Max. 2.9e7 cps.

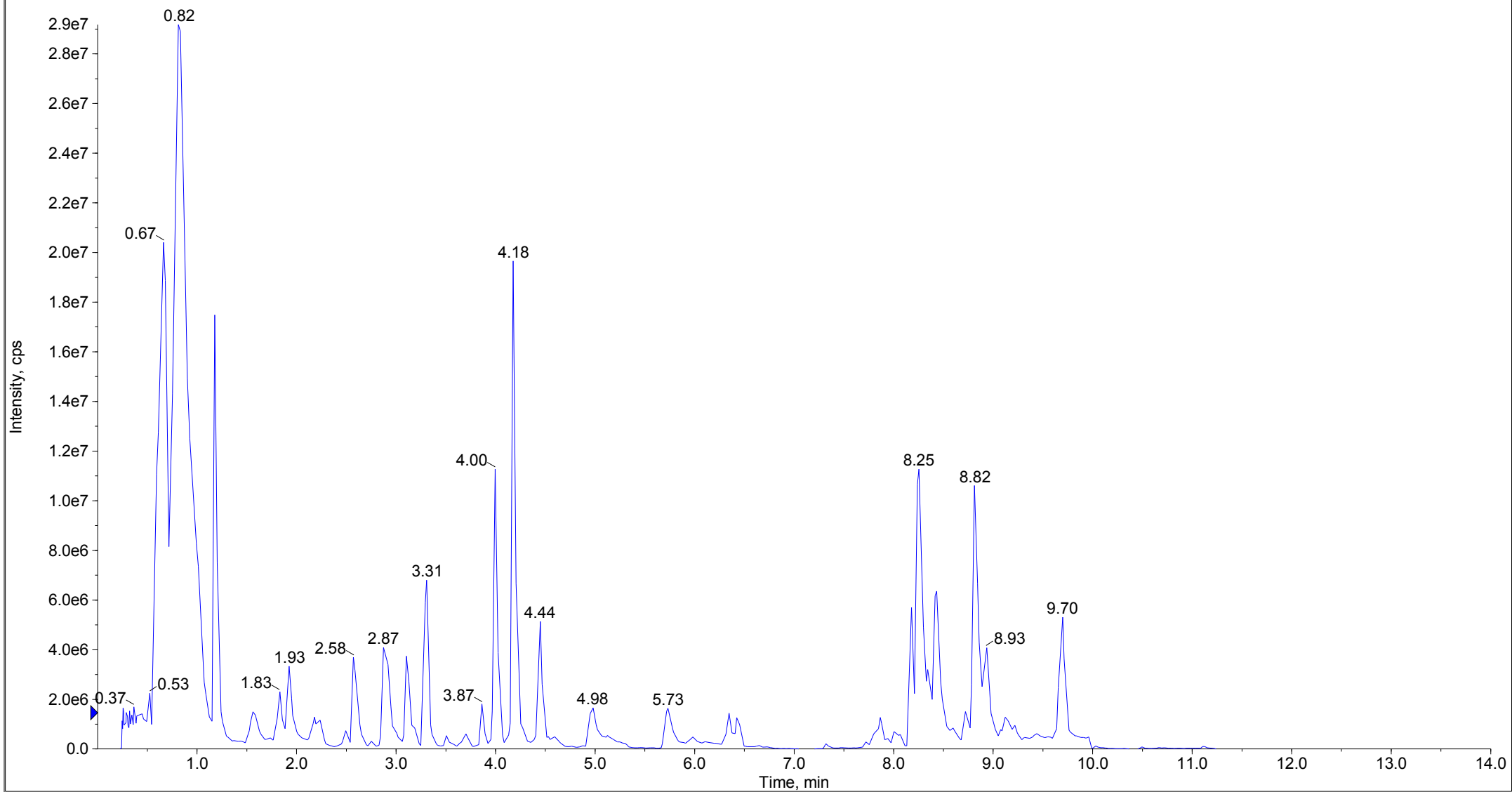

Supplement: Supplementary file 1 — Supplementary Information 1. [file 41598_2022_27019_MOESM1_ESM.zip › Additional file 1 The figure of total ion current/BSHC2-A19051334a_P.pdf]

TIC of -MRM (722 pairs): from Sample 23 (A19051337a\_N) of MWBMK-19-065\_12\_JS4500-2\_C02\_MWDB4.0\_ZW\_20200730.wiff (Turbo Spra...

Max. 3.6e7 cps.

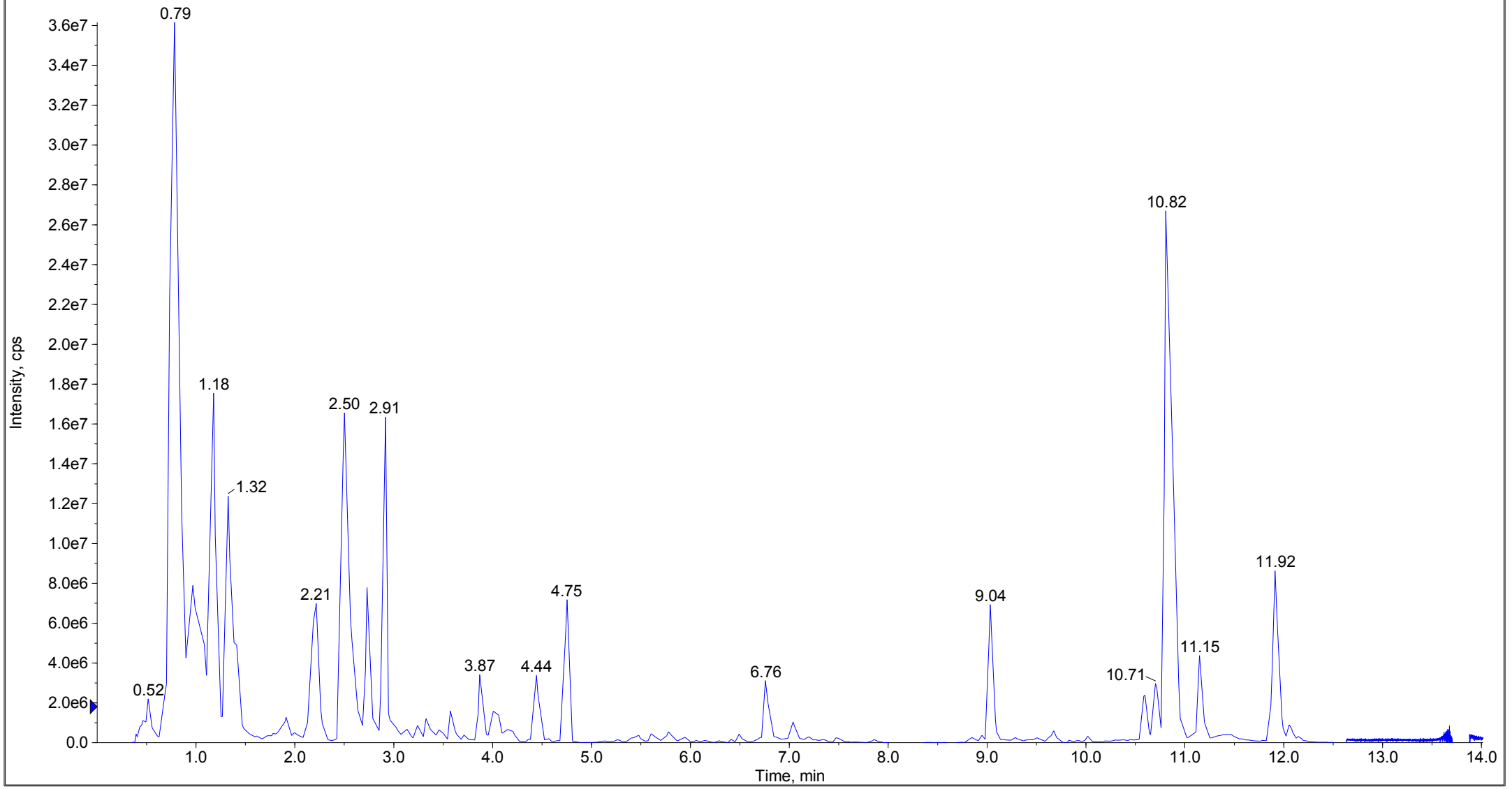

Supplement: Supplementary file 1 — Supplementary Information 1. [file 41598_2022_27019_MOESM1_ESM.zip › Additional file 1 The figure of total ion current/BSHC3-A19051337a_N.pdf]

TIC of +MRM (669 pairs): from Sample 5 (A19051337a\_P) of MWBMK-19-065\_12\_JS4500-2\_C02\_MWDB4.0\_ZW\_20200730.wiff (Turbo Spray...

Max. 3.3e7 cps.

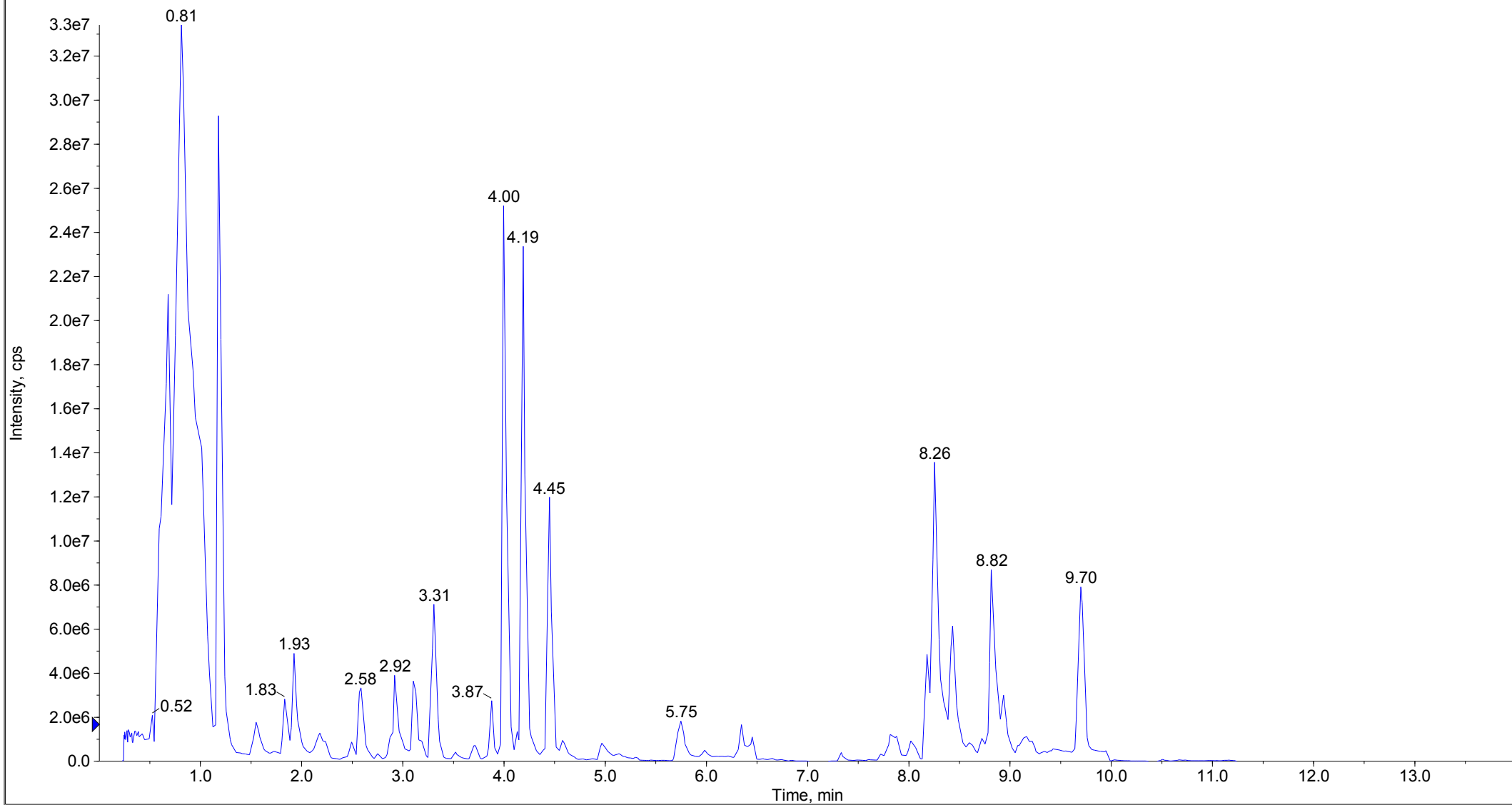

Supplement: Supplementary file 1 — Supplementary Information 1. [file 41598_2022_27019_MOESM1_ESM.zip › Additional file 1 The figure of total ion current/BSHC3-A19051337a_P.pdf]

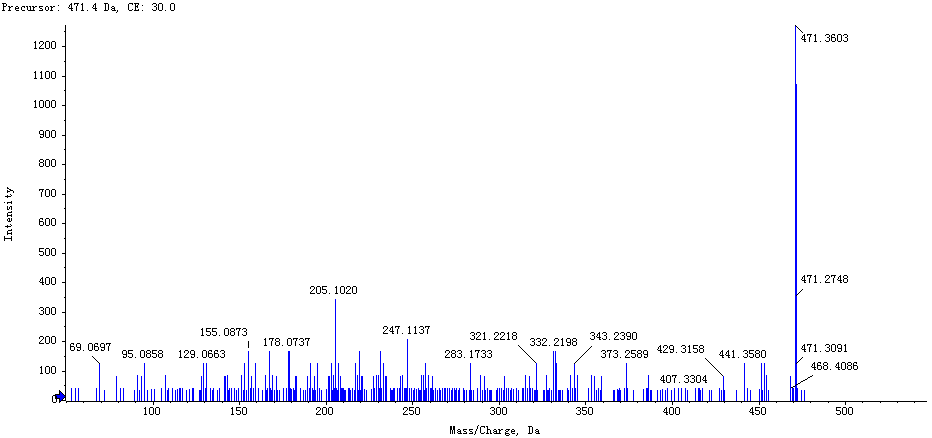

Supplement: Supplementary file 3 — Supplementary Information 3. [file 41598_2022_27019_MOESM3_ESM.zip › Additional file 3 Figures for the annotated MSMS spectra of highly differentiating metabolites/11-Keto-ursolic acid-╚2▌╞.png]

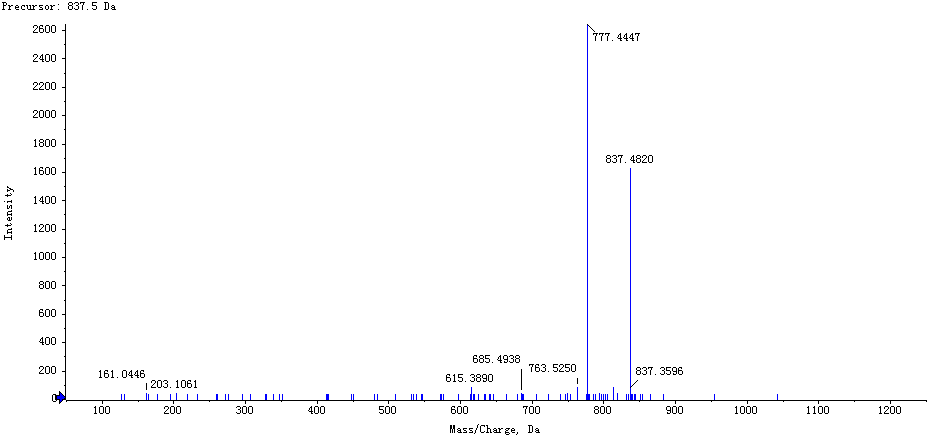

Supplement: Supplementary file 3 — Supplementary Information 3. [file 41598_2022_27019_MOESM3_ESM.zip › Additional file 3 Figures for the annotated MSMS spectra of highly differentiating metabolites/16-Keto-saikosaponin A-╚2▌╞╘φ▄╒.png]

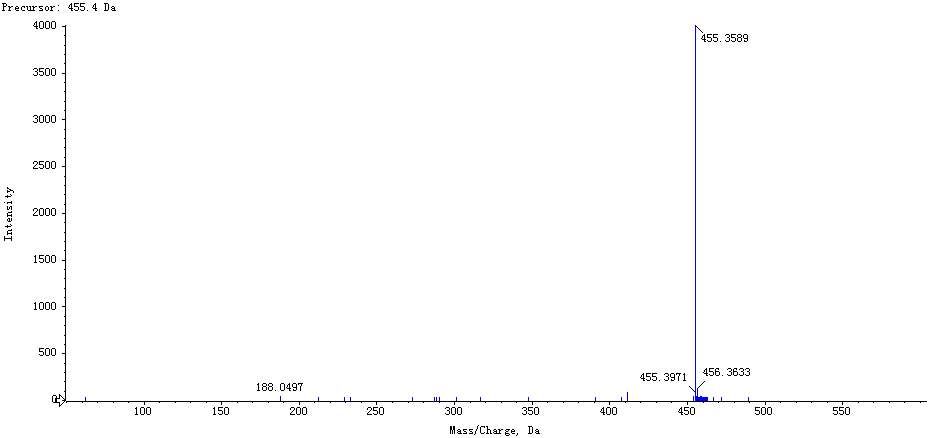

Supplement: Supplementary file 3 — Supplementary Information 3. [file 41598_2022_27019_MOESM3_ESM.zip › Additional file 3 Figures for the annotated MSMS spectra of highly differentiating metabolites/24,30-Dihydroxy-12(13)-enolupinol-╚2▌╞.png]

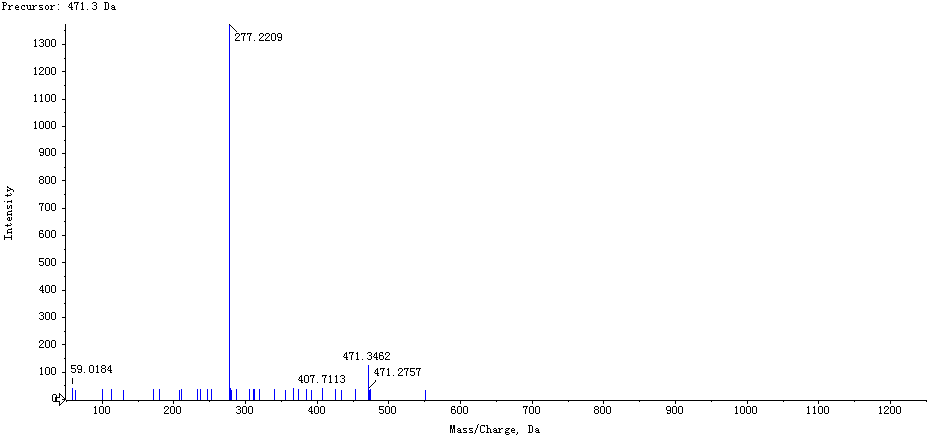

Supplement: Supplementary file 3 — Supplementary Information 3. [file 41598_2022_27019_MOESM3_ESM.zip › Additional file 3 Figures for the annotated MSMS spectra of highly differentiating metabolites/2a┴-Hydroxyursolic acid-╚2▌╞.png]

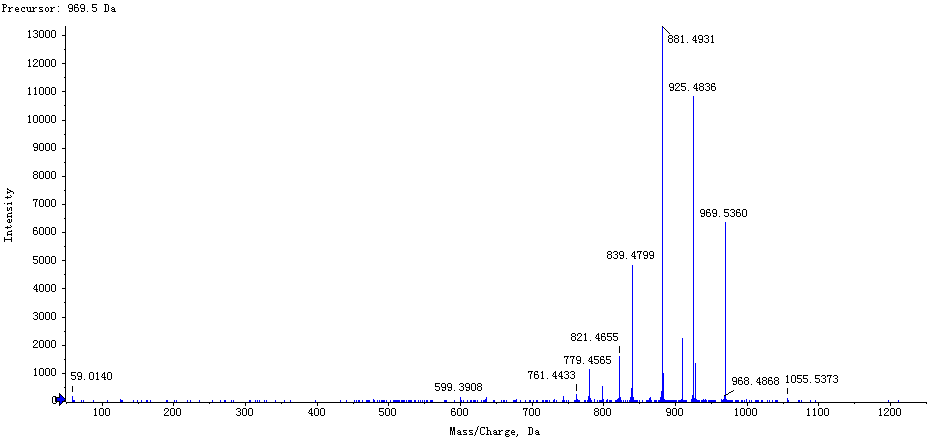

Supplement: Supplementary file 3 — Supplementary Information 3. [file 41598_2022_27019_MOESM3_ESM.zip › Additional file 3 Figures for the annotated MSMS spectra of highly differentiating metabolites/AcetylSaikosaponin F-╚2▌╞.png]

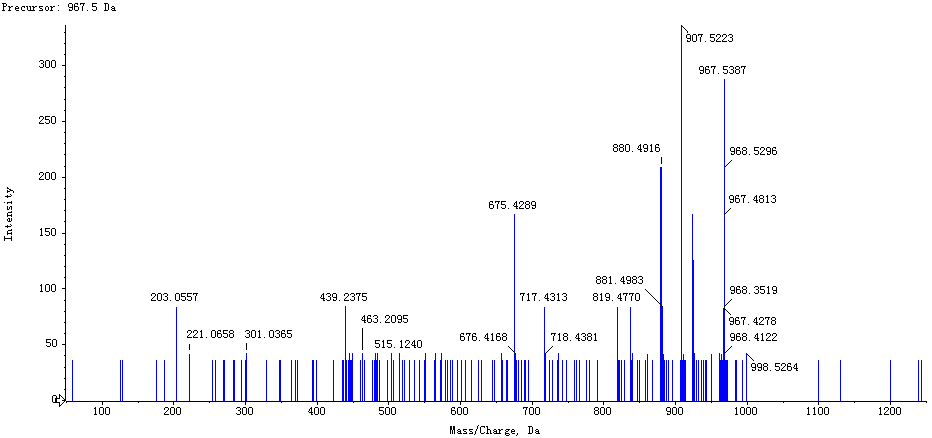

Supplement: Supplementary file 3 — Supplementary Information 3. [file 41598_2022_27019_MOESM3_ESM.zip › Additional file 3 Figures for the annotated MSMS spectra of highly differentiating metabolites/AcetylSaikosaponin I-╚2▌╞.png]

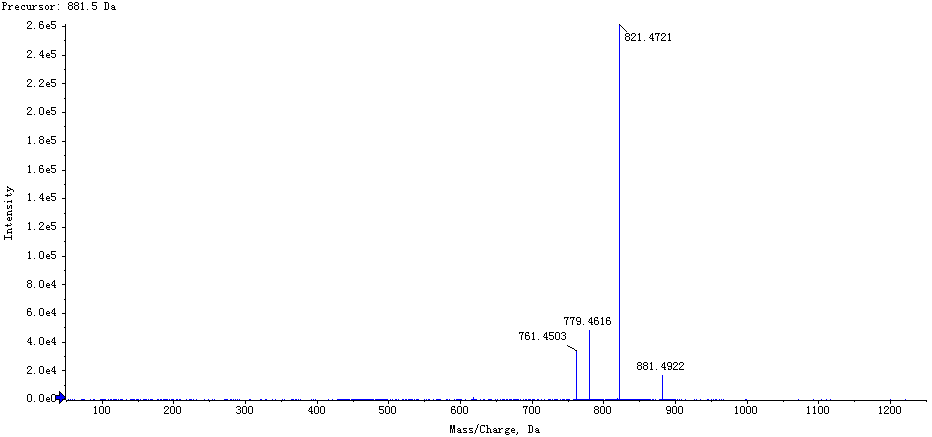

Supplement: Supplementary file 3 — Supplementary Information 3. [file 41598_2022_27019_MOESM3_ESM.zip › Additional file 3 Figures for the annotated MSMS spectra of highly differentiating metabolites/Acetylsaikosaponin A-╚2▌╞.png]

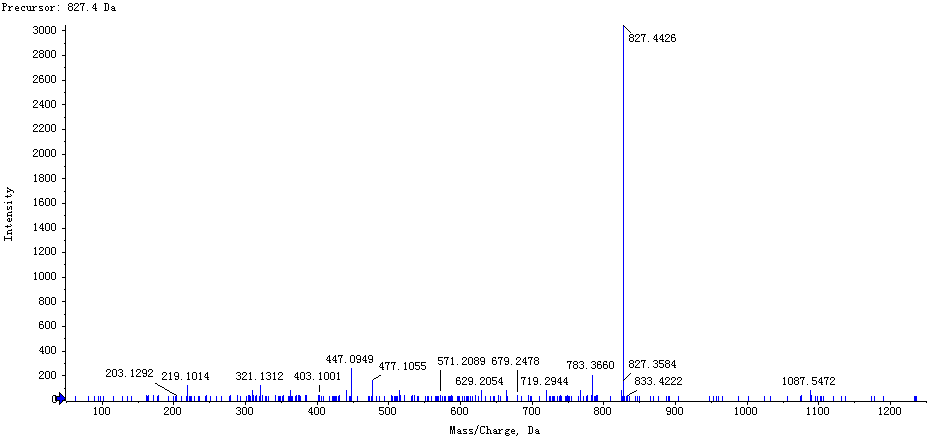

Supplement: Supplementary file 3 — Supplementary Information 3. [file 41598_2022_27019_MOESM3_ESM.zip › Additional file 3 Figures for the annotated MSMS spectra of highly differentiating metabolites/Centellasaponin B-╚2▌╞.png]

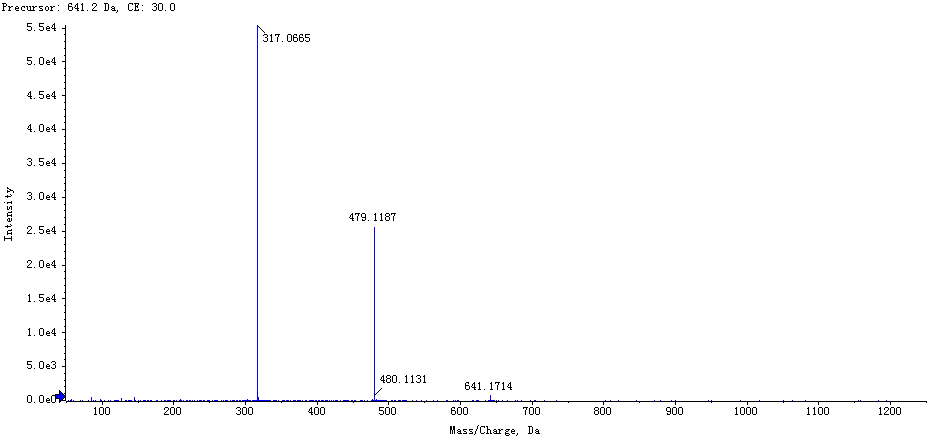

Supplement: Supplementary file 3 — Supplementary Information 3. [file 41598_2022_27019_MOESM3_ESM.zip › Additional file 3 Figures for the annotated MSMS spectra of highly differentiating metabolites/Isorhamnetin-3,7-O-diglucoside-╗╞═¬.png]

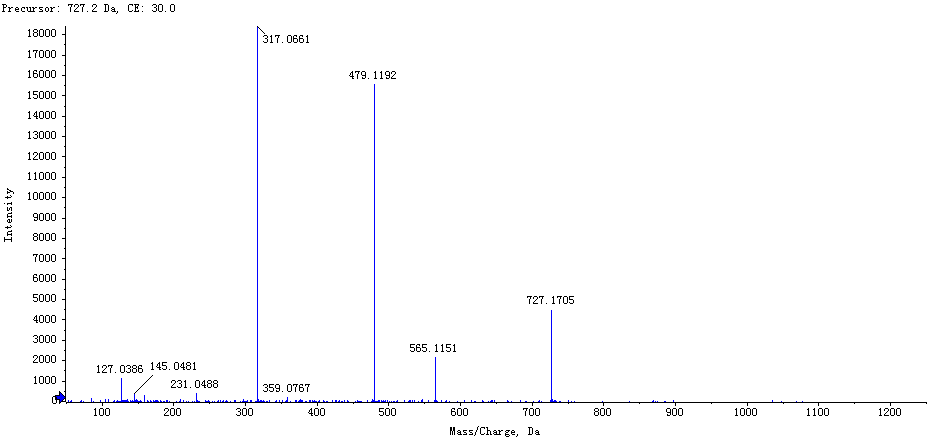

Supplement: Supplementary file 3 — Supplementary Information 3. [file 41598_2022_27019_MOESM3_ESM.zip › Additional file 3 Figures for the annotated MSMS spectra of highly differentiating metabolites/Isorhamnetin-3-O-(6''-malonylglucoside)-7-O-glucoside-╗╞═¬.png]

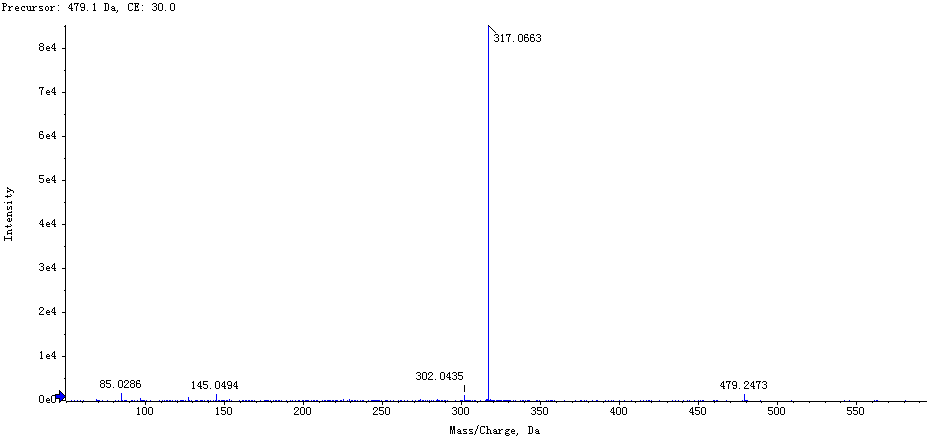

Supplement: Supplementary file 3 — Supplementary Information 3. [file 41598_2022_27019_MOESM3_ESM.zip › Additional file 3 Figures for the annotated MSMS spectra of highly differentiating metabolites/Isorhamnetin-3-O-Glucoside-╗╞═¬.png]

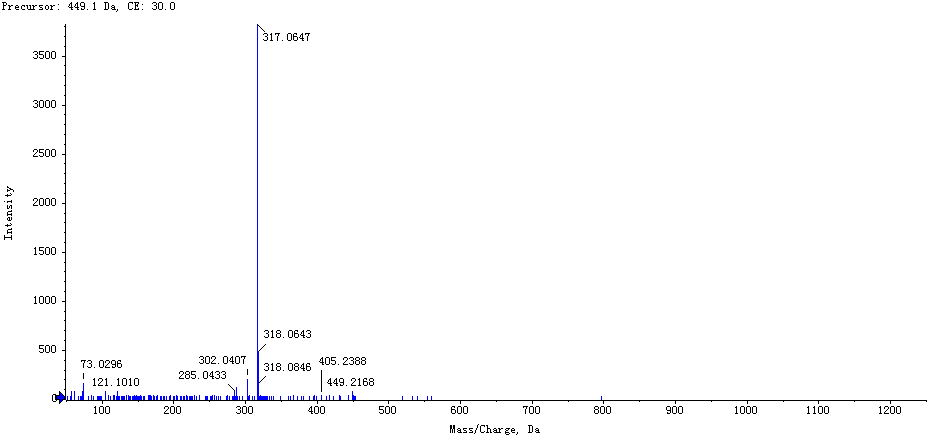

Supplement: Supplementary file 3 — Supplementary Information 3. [file 41598_2022_27019_MOESM3_ESM.zip › Additional file 3 Figures for the annotated MSMS spectra of highly differentiating metabolites/Isorhamnetin-3-O-arabinoside-╗╞═¬.png]

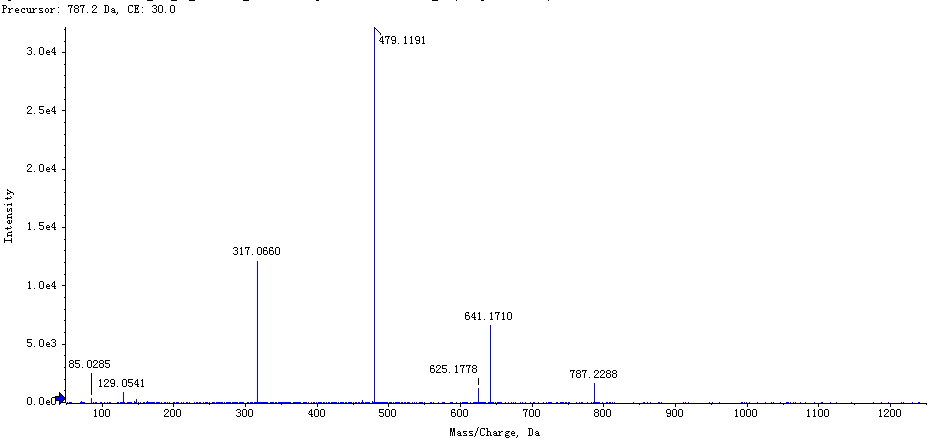

Supplement: Supplementary file 3 — Supplementary Information 3. [file 41598_2022_27019_MOESM3_ESM.zip › Additional file 3 Figures for the annotated MSMS spectra of highly differentiating metabolites/Isorhamnetin-3-O-rutinoside-4'-O-glucoside-╗╞═¬.png]

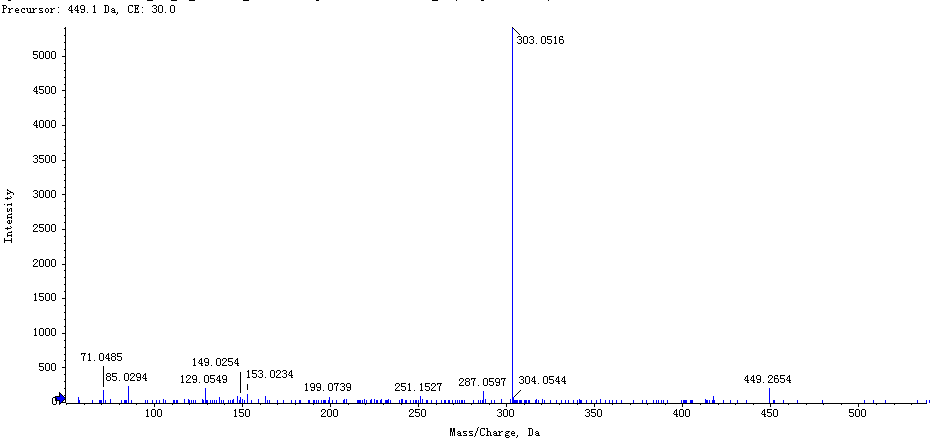

Supplement: Supplementary file 3 — Supplementary Information 3. [file 41598_2022_27019_MOESM3_ESM.zip › Additional file 3 Figures for the annotated MSMS spectra of highly differentiating metabolites/Kaempferol-4'-O-glucoside-╗╞═¬.png]

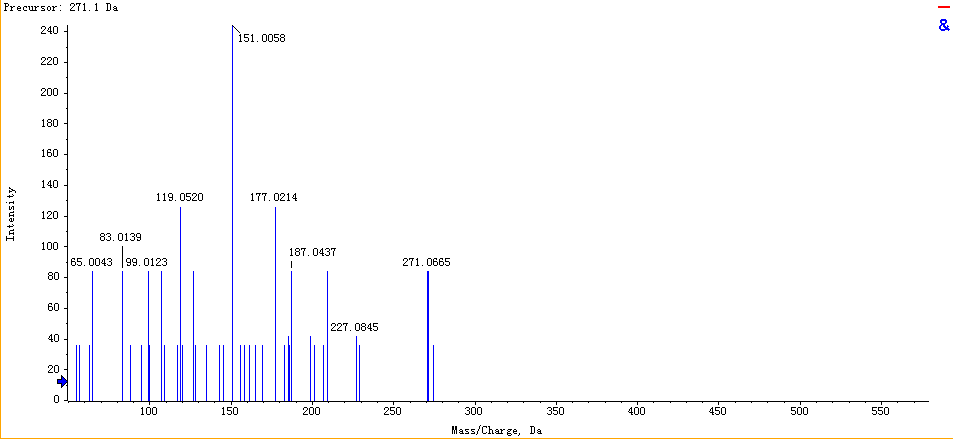

Supplement: Supplementary file 3 — Supplementary Information 3. [file 41598_2022_27019_MOESM3_ESM.zip › Additional file 3 Figures for the annotated MSMS spectra of highly differentiating metabolites/Naringenin (5,7,4'-Trihydroxyflavanone)-╗╞═¬.png]

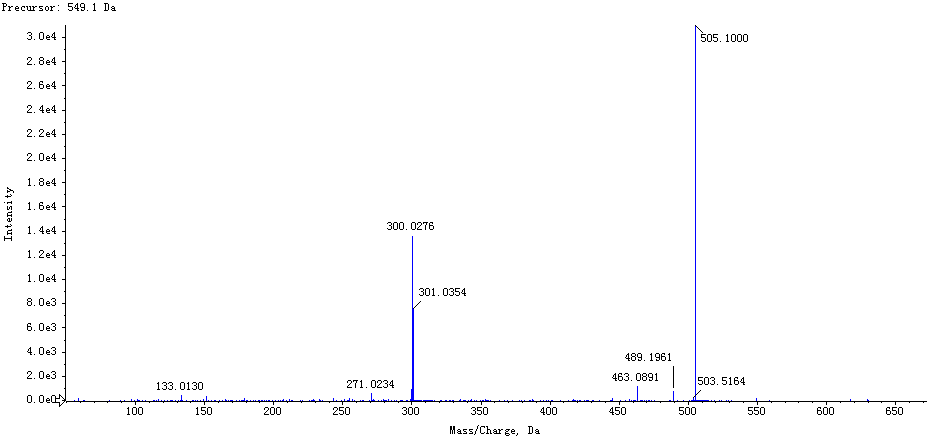

Supplement: Supplementary file 3 — Supplementary Information 3. [file 41598_2022_27019_MOESM3_ESM.zip › Additional file 3 Figures for the annotated MSMS spectra of highly differentiating metabolites/Quercetin-3-O-(6''-malonyl)glucoside-╗╞═¬.png]

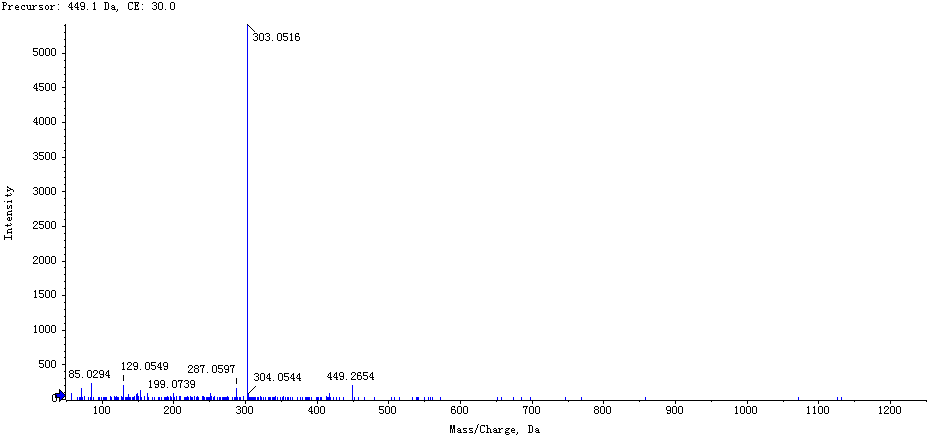

Supplement: Supplementary file 3 — Supplementary Information 3. [file 41598_2022_27019_MOESM3_ESM.zip › Additional file 3 Figures for the annotated MSMS spectra of highly differentiating metabolites/Quercetin-3-O-rhamnoside(Quercitrin)-╗╞═¬.png]

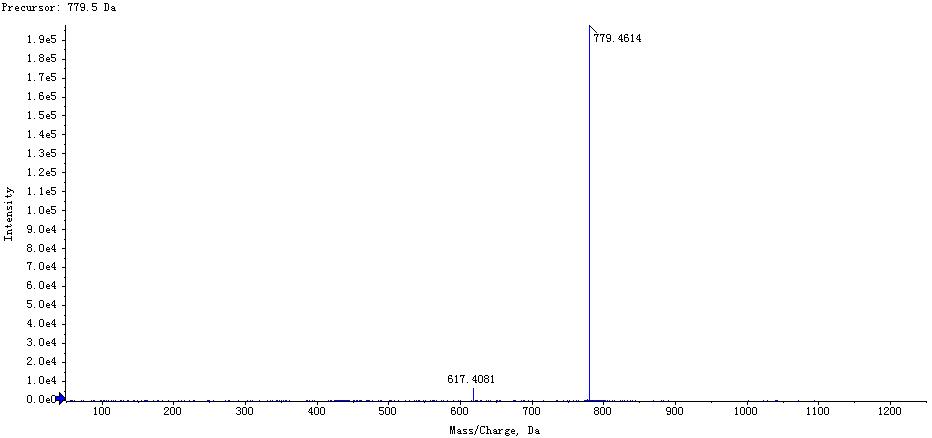

Supplement: Supplementary file 3 — Supplementary Information 3. [file 41598_2022_27019_MOESM3_ESM.zip › Additional file 3 Figures for the annotated MSMS spectra of highly differentiating metabolites/Saikosaponin A-╚2▌╞.png]

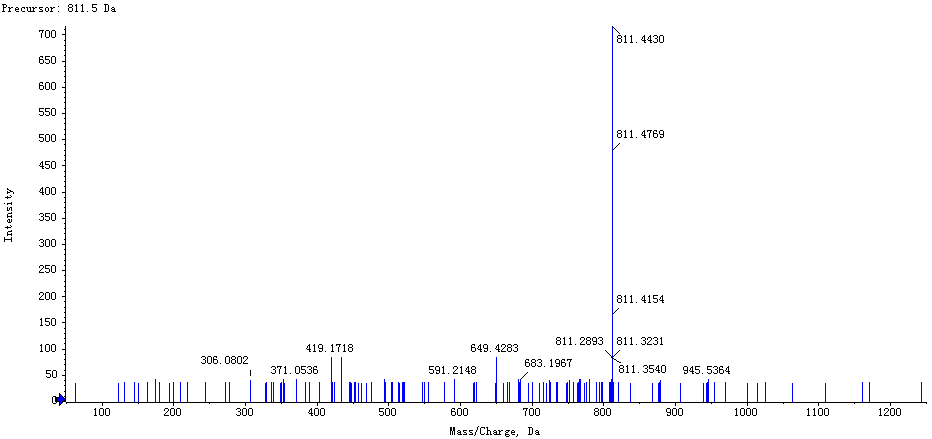

Supplement: Supplementary file 3 — Supplementary Information 3. [file 41598_2022_27019_MOESM3_ESM.zip › Additional file 3 Figures for the annotated MSMS spectra of highly differentiating metabolites/Saikosaponin B4-╚2▌╞.png]

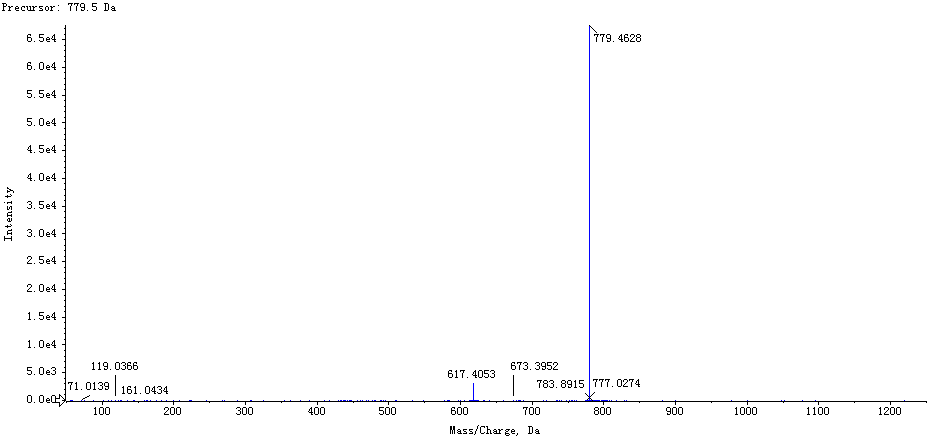

Supplement: Supplementary file 3 — Supplementary Information 3. [file 41598_2022_27019_MOESM3_ESM.zip › Additional file 3 Figures for the annotated MSMS spectra of highly differentiating metabolites/Saikosaponin D-╚2▌╞.png]

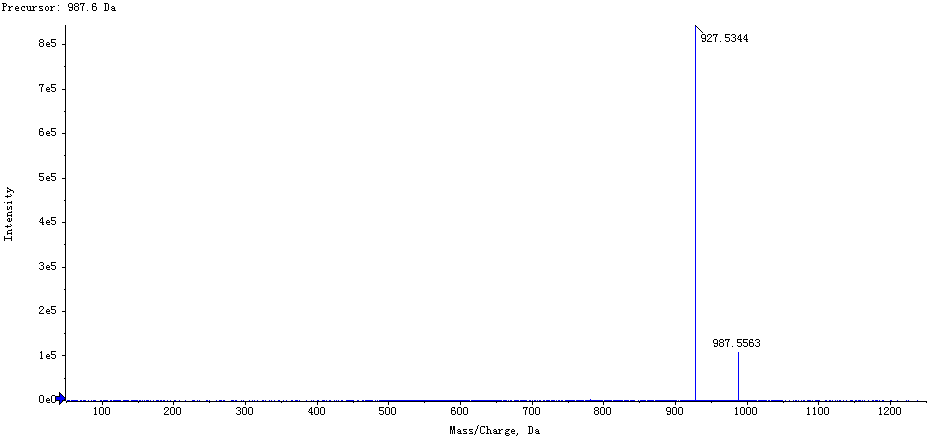

Supplement: Supplementary file 3 — Supplementary Information 3. [file 41598_2022_27019_MOESM3_ESM.zip › Additional file 3 Figures for the annotated MSMS spectra of highly differentiating metabolites/Saikosaponin F-╚2▌╞.png]

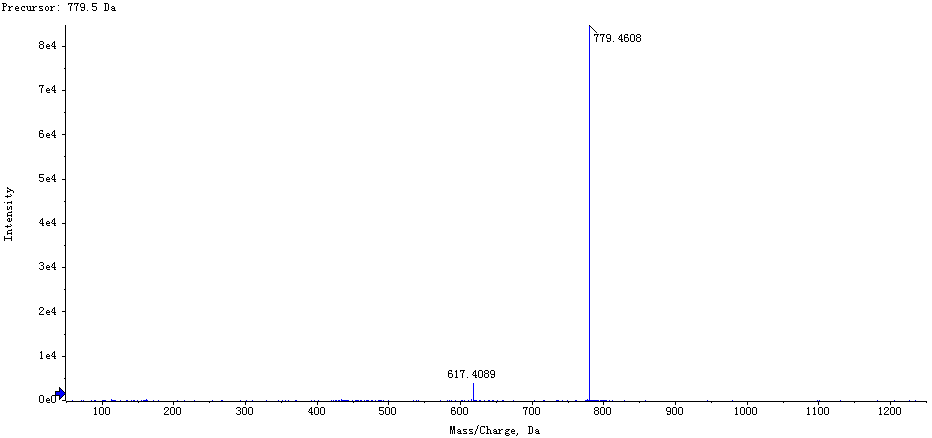

Supplement: Supplementary file 3 — Supplementary Information 3. [file 41598_2022_27019_MOESM3_ESM.zip › Additional file 3 Figures for the annotated MSMS spectra of highly differentiating metabolites/Saikosaponin G-╚2▌╞.png]

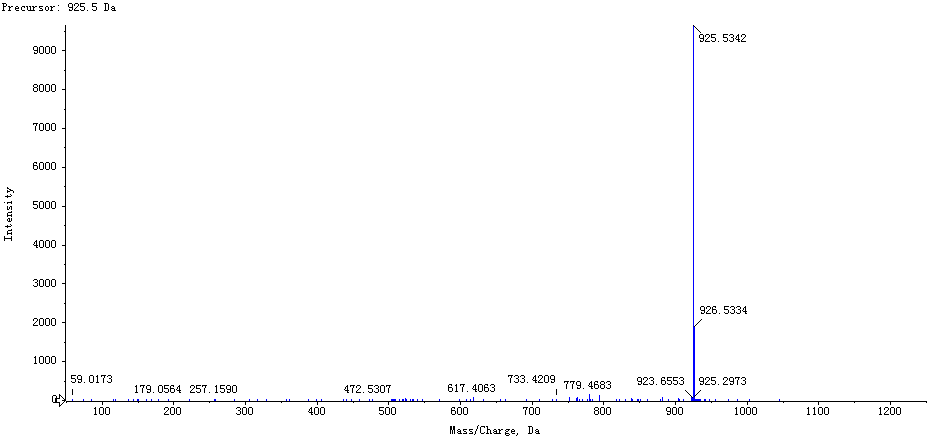

Supplement: Supplementary file 3 — Supplementary Information 3. [file 41598_2022_27019_MOESM3_ESM.zip › Additional file 3 Figures for the annotated MSMS spectra of highly differentiating metabolites/Saikosaponin I-╚2▌╞.png]
